# Glycolysis‐Derived Lactate Induces ACSL4 Expression and Lactylation to Activate Ferroptosis during Intervertebral Disc Degeneration

**DOI:** 10.1002/advs.202416149

**Published:** 2025-04-02

**Authors:** Kaiqiang Sun, Yangyang Shi, Chen Yan, Shunmin Wang, Linhui Han, Fudong Li, Ximing Xu, Yuan Wang, Jingchuan Sun, Zijian Kang, Jiangang Shi

**Affiliations:** ^1^ Department of Orthopedic Surgery Changzheng Hospital Navy Medical University Shanghai 200003 P. R. China; ^2^ Department of Orthopedics Naval Medical Center of PLA Shanghai 200052 P. R. China; ^3^ Department of Rheumatology and Immunology Shanghai Sixth People's Hospital Shanghai Jiao Tong University School of Medicine Shanghai 200003 P. R. China

**Keywords:** ACSL4, H3K18la, intervertebral disc degeneration, lactylation, metabolic reprogramming, SIRT3, therapy

## Abstract

The abnormal activation of the inflammatory microenvironment is frequently accompanied by metabolic changes that affect the development of various diseases. However, the relationship between metabolic reprogramming and intervertebral disc degeneration (IVDD) remains unclear. This study aims to reveal the metabolic changes in nucleus pulposus (NPCs) during IVDD and investigate the mechanism of glycolysis‐derived lactate on NPCs. Single‐cell RNA sequencing reveals that during IVDD, NPCs are characterized by excessively elevated glycolysis, and the resultant lactate causes the dysfunction of NPCs via ferroptosis activation. Mechanistically, lactate results in the transcription of Acyl‐CoA Synthetase Long Chain Family Member 4 (ACSL4) via promoting Histon H3K18 lactylation. Interestingly, lactate can also increase the lactylation of ACSL4 at K412 site. In addition, lactate‐induced decreased expression of Sirtuin‐3 (SIRT3), and further cause the elevation of ACSL4 lactylation. Finally, animal experiments demonstrate that inhibiting glycolysis through gene silencing with adenoviral‐associated viruses 9 (AAV9)‐si‐*Ldha* or chemical treatment using 2‐deoxy‐d‐glucose can suppress lactate production and lactylation, thereby ameliorating ferroptosis and NPC dysfunction. The findings of this study indicate that lactate plays a crucial role in IVDD by activating ferroptosis and that interventions aimed at lactate production can offer a potential therapeutical option for patients with IVDD.

## Graphical abstract

Glycolysis‐derived lactate activates nucleus pulposus cell ferroptosis via Histon H3K18la‐mediated ACSL4 transcription and ACSL4 lactylation and aggravate intervertebral disc degeneration. Inhibiting glycolysis via gene silencing or chemical intervention reduces the production of lactate and ameliorates ferroptosis activation and nucleus pulposus dysfunction during intervertebral disc degeneration.

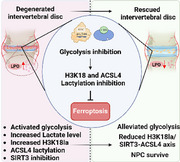

## 1 Introduction

Intervertebral disc degeneration (IVDD) is an extremely common chronic degenerative condition affecting individuals across all ages, with over 560 million people worldwide impacted. It is a leading cause of disability, resulting in significant healthcare costs, reduced productivity, and diminished quality of life, thereby imposing a substantial socioeconomic burden on global healthcare systems.^[^ ^1^ ^]^ Low back pain (LBP) is the most common symptom, affecting >80% of people worldwide during their lifetime.^[^ ^2^ ^]^ Clinical treatment of IVDD mainly includes conservative and surgical treatments.^[^ ^2^ ^]^ However, the pathophysiological process of IVDD cannot be retarded using either approach, and surgery‐related complications severely affect the quality of life of patients.^[^ ^3^ ^]^

Nucleus pulposus cell (NPC) dysfunction is a critical feature of IVDD.^[^ ^4^ ^]^ Healthy NP tissue is marked by sufficient extracellular matrix (ECM) and properly dispersed NPCs.^[^ ^5^ ^]^ However, due to mechanical stimuli or inflammatory responses, the metabolic homeostasis of the ECM is disturbed, characterized by decreased synthesis of type II collagen and aggrecan and increased secretion of type I/III collagen, matrix metalloproteases (MMPs), and A Disintegrin and Metalloproteinase with Thrombospondin motifs (ADAMTSs).^[^ ^6^ ^]^ Thus, increased type I/III collagen and MMP2/3/13 levels shift the phenotype of NP tissue to a fibroblast‐like phenotype.^[^ ^7^, ^8^ ^]^ However, despite the high prevalence of IVDD, no effective disease‐modulating therapies for IVDD is available because of the lack of an in‐depth understanding of disease pathogenesis.

Energy metabolism is an important modulator of cellular function, and abnormal energy supply often contributes to the onset of various diseases.^[^ ^9^ ^]^ IVDD and osteoarthritis (OA) have many pathophysiological similarities; both are age‐related degenerative dysfunctions characterized by low‐grade inflammation and ECM metabolism disequilibrium.^[^ ^10^, ^11^ ^]^ The similarities between IVDD and OA primarily depend on NPCs and chondrocytes.^[^ ^11^ ^]^ Chondrocytes in joints reportedly undergo metabolic remodeling during OA frequently.^[^ ^12^, ^13^ ^]^ Owing to the hypoxic environment, chondrocytes receive energy primarily by relying on glycolysis, making glucose their primary metabolic fuel.^[^ ^14^ ^]^ However, the supply of oxygen and nutrients is disturbed during OA, thereby enhancing glycolysis.^[^ ^15^ ^]^ In addition, continuous activation of glucose transporter 1 (GLUT1) can induce proteoglycan depletion in the cartilage by producing advanced glycation end products (AGEs).^[^ ^16^, ^17^ ^]^ Similarly, the major energy source for NPCs is glucose glycolysis.^[^ ^18^ ^]^ Hypoxia‐inducible factor (HIF‐1α) is highly expressed in NPCs to adapt to hypoxic environments. Kim et al.^[^ ^19^ ^]^ revealed that constitutively activating HIF‐1α signaling pathway play a protective role against IVDD by enhancing glycolysis. However, Wang et al.^[^ ^20^ ^]^ recently reported upregulated HIF‐1α signaling in degenerative human and mouse IVD, and that abnormal activation of HIF‐1α signaling increased the risk of age‐dependent IVDD, accompanied by intensifying glycolytic metabolism and damaged mitochondrial function. Thus, the exact effects of glycolysis on IVDD remain unknown.

Therefore, the present study aimed to reveal the changes in glycolytic remodeling in NPCs during IVDD and explore the regulatory mechanism of glycolysis‐derived lactate on NPCs. The results of this study provide new insights and therapeutic targets for the treatment of IVDD from the perspective of metabolic remodeling.

## 2 Results

### 2.1 Single‐Cell Transcriptome Profiling Reveals Enhanced Glycolysis in NPCs during IVDD

**Figure 1:**
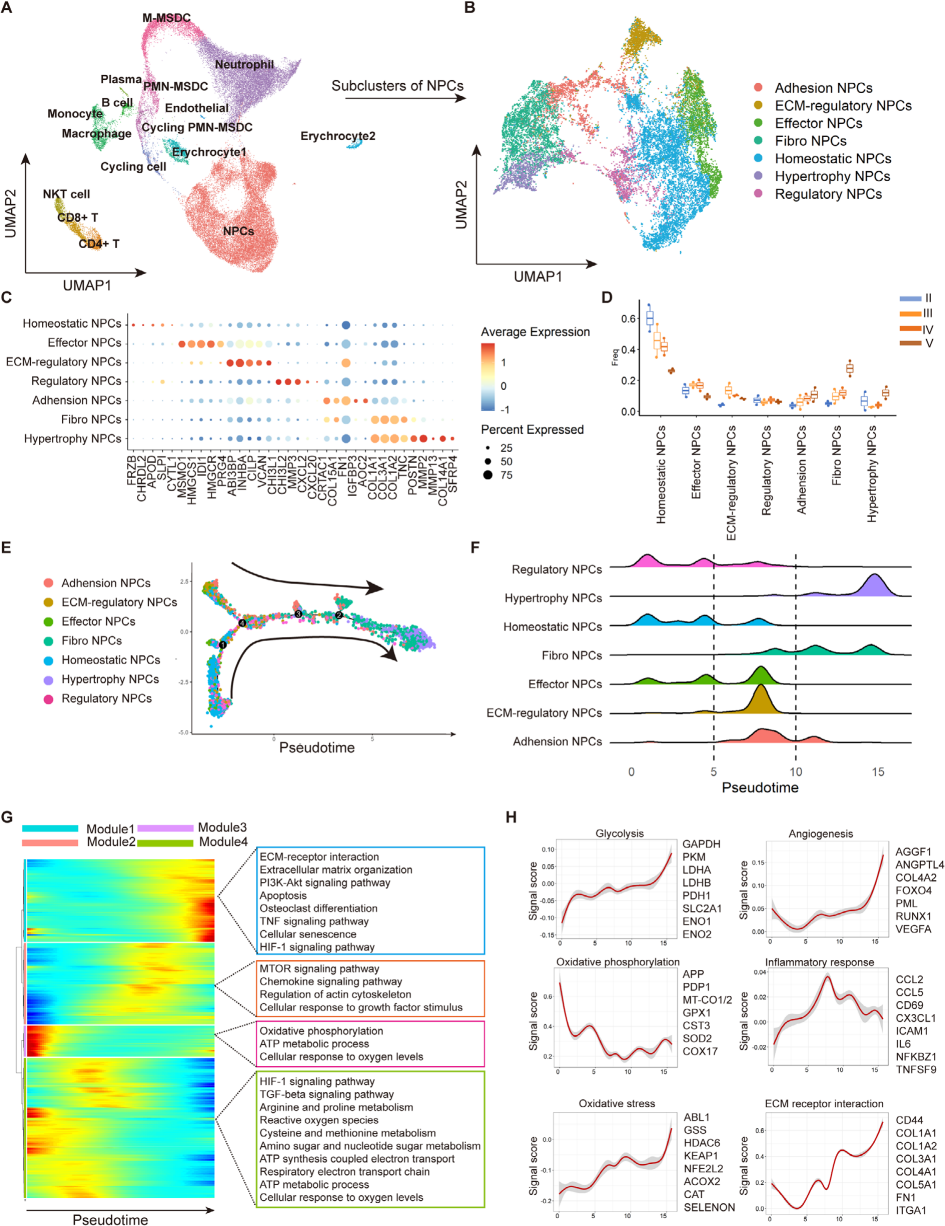
Single‐cell transcriptome profiling of human IVD tissue reveals activated glycolysis of NPCs during IVDD. A) UMAP visualizing human NPCs as seven different clusters within human IVD tissue after unsupervised clustering. Each plot indicated one single cell and colored based on different cell subclusters. B) UAMP of expression pattern of the DEG that best identified each NP cell subcluster. Values indicated are log‐normalized counts per cell. C) Dot plot of highest DEGs for each major NP cell subcluster. D) Box plot showing the proportions of NP subclusters in IVD patients with different severity of IVDD. E) Trajectory differentiation diagram colored by cell types and differentiated states. Cells within initial state were homeostatic NPCs. F) The detailed distribution of different NPCs in trajectory plot during differentiation. G) Branch trajectory heatmap of the DEGs revealed the gene alterations under different cell differentiation states. The main functions of genes in each cluster are also shown. H) Signature score of the representative pathways for energy metabolism of human NP cells. IVD: Intervertebral disc; IVDD: Intervertebral disc degeneration; NPCs: Nucleus pulposus cells; DEG: Differentially expressed gene; UMAP: Uniform manifold approximation and projection.

Single‐cell sequencing enables precise analysis of individual cells, revealing cellular heterogeneity, dynamic states, and molecular functions. It provides critical insights into disease mechanisms, tissue regeneration, and metabolic remodeling in IVDD. Therefore, to comprehensively explore the glucose metabolic changes in NPCs during IVDD, we searched the published Single‐cell RNA sequencing (ScRNA‐seq) data, and we chose the database (GSE165722)^[^ ^21^ ^]^ because it contains IVD tissues from patients with varying degrees of degeneration (Grade II–V based on Pfirrmann score of T2‐weighed MRI). The cellular composition was analyzed via unbiased clustering across all cells using principal component analysis (PCA) and visualized using Uniform Manifold Approximation and Projection (UMAP). Sixteen major clusters representing different cell types were identified, with NPCs accounting for the majority (Figure S1A,B, Supporting Information). Human NPCs were primarily classified into seven different clusters: homeostatic, ECM‐regulatory, adhesion, effector, regulatory, fibrotic, and hypertrophic NPCs (**Figure** **1**A–C). Homeostatic NPCs accounted for the major proportion in grade II IVD tissue and decreased with the severity of IVDD, whereas adhesion, hypertrophic, and fibrotic NPCs showed an increasing tendency in IVD tissue from grades II to V (Figure 1D).

The original study provided a broad cellular map of the intervertebral disc (IVD) tissue, including NPCs and other cell types. In contrast, our single‐cell analysis focuses specifically on the functional and metabolic dynamics within degenerating NPCs. To explore the molecular changes in IVDD‐involved NPCs, we used Monocle2 to reconstruct the differentiation trajectory of the NPCs lineage in pseudo‐time and found that the overall differentiation trajectory of disc degeneration was revealed by starting from homeostatic NPCs and branching toward fibrotic and hypertrophic NPCs (Figure 1E). By analyzing each subpopulation in the pseudo‐time trajectory, we found that adhesion, ECM‐regulatory, effector, and homeostatic NPCs were in the starting position, whereas fibrotic and hypertrophic NPCs were present in the terminal disease stage (Figure 1F). To explore gene expression dynamics along pseudo‐time trajectories, we explored differentially expressed genes (DEGs) that constantly increased or decreased during IVDD and clustered them DEGs on their expression patterns (Figure 1G). In this manner, we identified 2358 IVDD‐associated genes and grouped them into four modules based on their variation among cell clusters. Modules 1 and 2 were upregulated at a later stage of the disease, and Modules 3 and 4 were upregulated at an earlier stage of the disease. Subsequent Gene ontology (GO) and Kyoto Encyclopedia of Genes and Genomes (KEGG) analyses revealed that Module 1 was associated with pathological changes in IVDD, such as “ECM‐receptor interaction,” “Ferroptosis,” “Cellular senescence,” and “glycolysis” (Figure 1G). Module 2 also indicated disease progression and was enriched in “mammalian target of rapamycin (mTOR) signaling pathway,” “Chemokine signaling pathway,” and “Regulation of actin cytoskeleton” (Figure 1G). Modules 3 and 4 play a role during the initiation stage and have elevated function involved in energy metabolism such as “Oxidative phosphorylation,” “ATP metabolic process,” “adenosine triphosphate (ATP) synthesis coupled electron transport,” and “respiratory electron transport chain” (Figure 1G). Our findings indicate that energy metabolism disorders may be a prerequisite for the phenotypic transition of homeostatic NPCs to fibrotic NPCs during IVDD.

To comprehensively investigate changes in energy metabolism during disc degeneration, we created a signature score of energy metabolism pathways. Signature scores for known physiological pathways were constructed to estimate disease progression. We found that the glycolysis score elevated and the oxidative phosphorylation score reduced during disc degeneration (Figure 1H). Other pathological pathway scores were also elevated during IVDD, such as angiogenesis and oxidative stress, supporting the degenerative changes in the NPCs of intervertebral disc (Figure 1H). Changes in the expression of glycolysis‐related genes, such as glyceraldehyde‐3‐phosphate dehydrogenase (GAPDH), lactate dehydrogenase A (LDHA), Enolase 1 (ENO1), and pyruvate kinase M (PKM), were also observed (Figure S2, Supporting Information). These results suggest that the cellular metabolic shift from oxidative phosphorylation to glycolysis is associated with IVDD progression.

### 2.2 Degenerated NPCs Is Accompanied by Activated Glycolytic Metabolism

**Figure 2:**
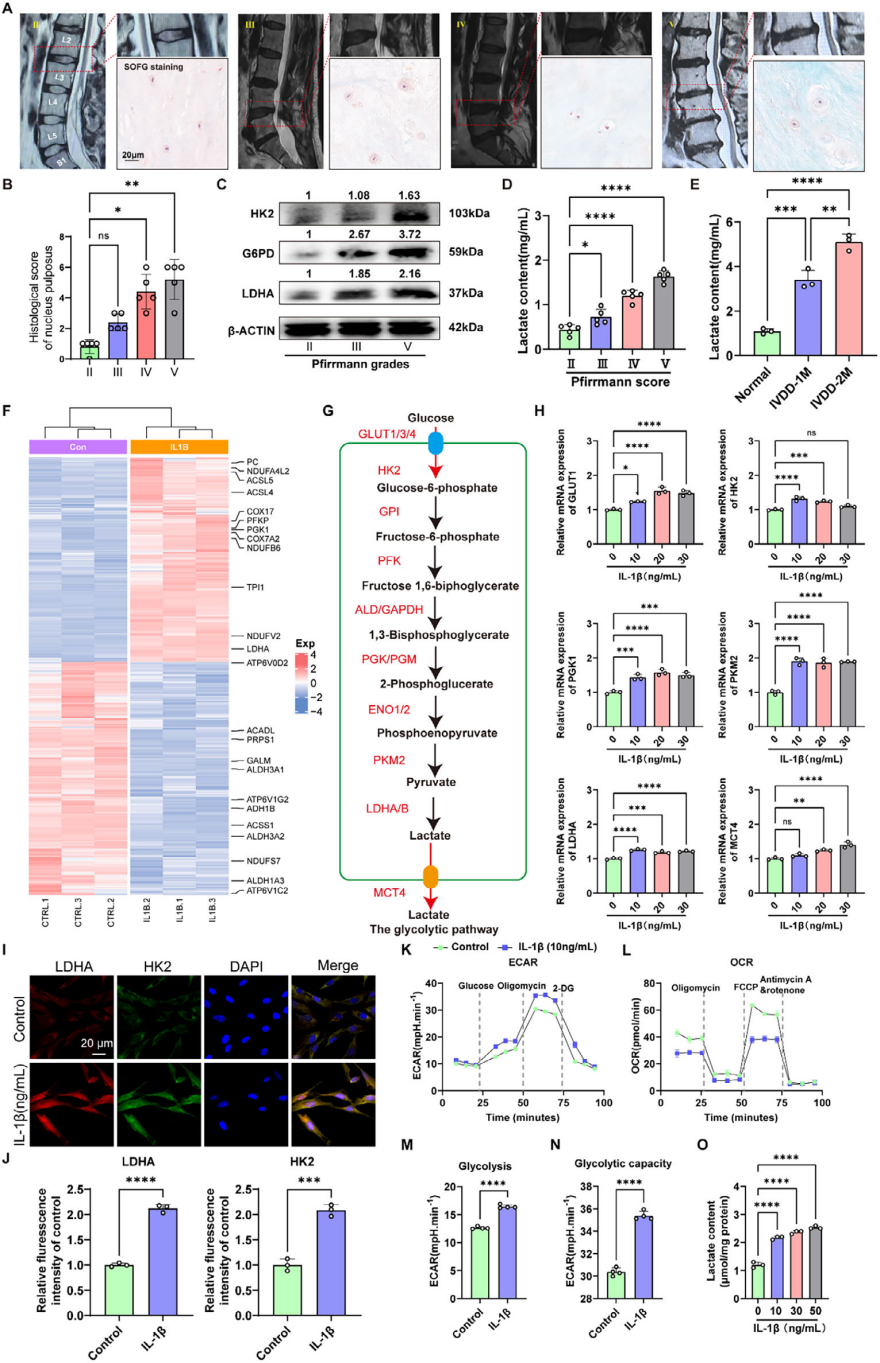
Degenerated human NP tissue is accompanied by enhanced glycolysis A) SOFG staining of human intervertebral disc NP tissue with different Pfirrmann score based on typical T2‐weighed MRI. Scale bar = 20 µm. B) Histological score using the new proposed grading system showed that the histological score of NP tissue was positive with the grade based on T2‐weighed MRI. C) Representative blot from Western blot for HK2, G6PD, and LDHA in human NP tissue with different degeneration grade. D) The lactate content in human NP tissue with different degeneration grade (*n* = 5). E) The lactate content in mouse IVD tissue at 0, 1 m, and 2 m of IVDD surgery (*n* = 3). F) Heat map suggesting the differentially expressed genes related to glycolysis in IL‐1β‐treated human NPCs. G) Illustration of glycolysis process and related rate‐limiting enzymes. H) RT‐qPCR results of the expression of glycolysis‐related genes in human NP cells treated by IL‐1β in a dose‐dependent manner. I) Representative images of IF staining for HK2 and LDHA in human NPCs with or without IL‐1β. Scale bar = 20 µm. J) Quantification of IF staining for LDHA and HK2. K) ECAR measurement in glycolysis stress test of human NPCs treated by IL‐1β. L) OCR measurement in MitoStress test of human NP cells treated by IL‐1β. M,N) Quantification of glycolysis and glycolytic capacity at one timepoint in glycolysis stress test. O) The lactate content in NPCs treated by IL‐1β in a dose‐dependent manner. SOFG: Safranin O‐Fast Green; NP: Nucleus pulposus; HK2: Hexokinase 2; G6PD: Glucose‐6‐phosphate dehydrogenase; LDHA: Lactate dehydrogenase A; ECAR: Extracellular acidification rate; OCR: Oxygen consumption rate; RT‐qPCR: RNA reverse transcription, and quantitative real‐time polymerase chain reaction. All data are shown as the mean ± SD. Two‐tailed unpaired Student's *t*‐tests (C, G, I, L, M, and N) and one‐way analysis of variance (ANOVA) were used followed by Tukey's post hoc test (D) to determine the statistical significance. *P < 0.05, **P < 0.01, ***P < 0.001, ****P < 0.0001.

Histologically, we first evaluated the expression of glycolysis‐related proteins, including hexokinase 2 (HK2), LDHA, and glucose‐6‐phosphate dehydrogenase (G6PD), in human intervertebral NP tissues. According to T2‐weighed MRI, human NP tissues were divided four groups, and SOFG staining was sued to evaluate the degeneration state histologically (**Figure** **2**A). Furthermore, we evaluated the histological score using the new proposed grading system,^[^ ^22^ ^]^ and the results showed that the histological score of NP tissue was positively with the grade based on T2‐weighed MRI (Figure 2B). We found that the expression of HK2, G6PD, and LDHA in NP tissues increased with IVDD severity (Figure 2C). In addition, the lactate content in NP tissue also showed a positive relationship with Pfirrmann grade and the concentrate of lactate was more than 1 mg mL^−1^ (about 11 × 10^−3^ m) when the Pfirrmann score of intervertebral disc was higher than grade IV (Figure 2D). Furthermore, we established mice model of IVDD using a needle‐injured tail and found that the lactate content in the mouse NP tissue increased gradually in accordance with the degeneration (Figure 2E). These results showed that glycolysis was excessively activated during IVDD.

Abnormal inflammatory response is a critical characteristic of IVDD.^[^ ^23^ ^]^ Several critical genes are involved in glycolysis, including glucose transporters 1/3/4 (GLUT1/3/4), HK2, phosphofructokinase (PFK), phosphoglycerate Kinase 1 (PGK1), pyruvate kinase M2 (PKM2), LDHA, and monocarboxylate transporter 4 (MCT4).^[^ ^24^ ^]^ We performed bulk mRNA sequencing with human NPC degeneration model using by Interleukin 1β (IL‐1β, 10 ng mL^−1^). Heatmap analysis showed the highly expressed glucose metabolism‐related critical genes in IL‐1β‐treated human NPCs (Figure 2F). For example, Il‐1β substantially upregulated the expression of glycolysis‐related genes, including PFK, PGK1, and LDHA, and the critical rate‐limiting enzyme for glycolysis (Figure 2F,G). RT‐qPCR analysis indicated that the degenerated NPCs expressed higher levels of glycolytic genes (Figure 2H). Consistently, immunofluorescence (IF) staining experiment also showed that the administration of IL‐1β significantly promoted the expression of LDHA and HK2 in human NPCs (Figure 2I,J). To dynamically uncover the inflammatory stimulus‐induced metabolic changes, we performed live‐cell metabolic assays and measured glycolytic (extracellular acidification rate, ECAR) and mitochondrial (oxygen consumption rate, OCR) respiration fluxes. At basal stage, IL‐1β showed no significant effect on ECAR (Figure 2K); however, after glucose was administrated into the cells, glycolysis was evidently enhanced by IL‐1β (Figure 2L–O). Nevertheless, mitochondrial respiration of human NPCs was suppressed with the treatment of IL‐1β (Figure 2L–O). In addition, IL‐1β dose‐dependently increased the production of lactate in NPCs (Figure 2O). These results suggest a shift from aerobic to anaerobic metabolism during inflammation‐triggered degeneration of human NPCs.

**Figure 3:**
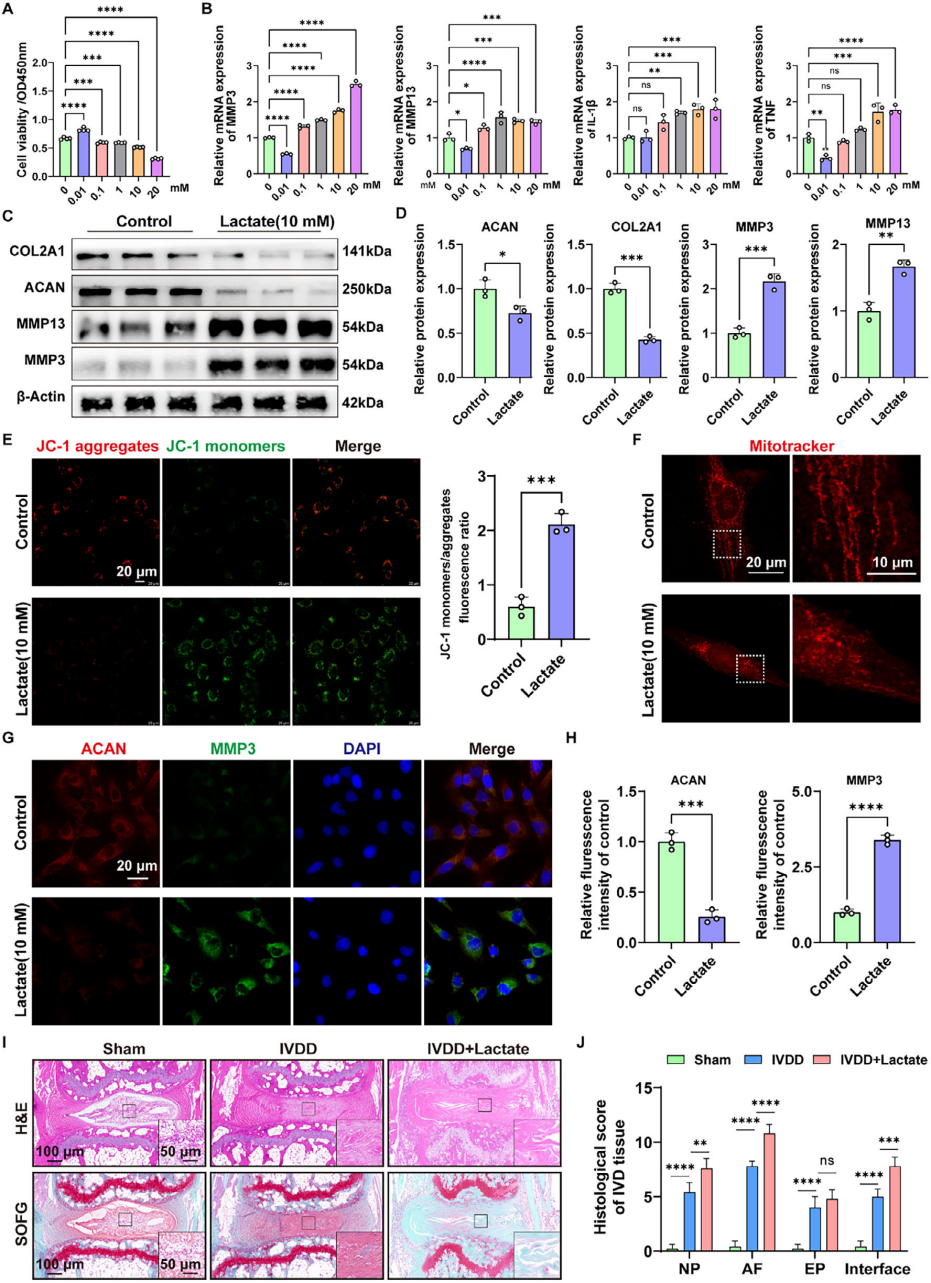
Excessively increased lactate contributes to the degradation of ECM in NPCs in vitro. A) The effect of lactate with different concentrations on the cell viability of human primary NPCs (*n* = 4). B) RT‐qPCR results of the expression of IVDD‐related genes (MMP3, MMP13, IL‐1β, and TNFα) in human primary NPCs treated by lactate in a dose‐dependent manner (*n* = 3). C) Western blot results of the expression of IVDD‐related proteins (MMP3, MMP13, ACAN, and COL2A1) in human primary NPCs treated by lactate (10 × 10^−3^ m) (*n* = 3). D) Quantitation of Western blot results. E) Representative image of mitochondrial membrane potential in human primary NPCs with or without the treatment of lactate for 24 h, as well as the quantitative result of the ratio of JC‐1 monomers to aggregates (*n* = 3). F) Representative image of IF staining for mitochondria suggested that the administration of lactate significantly damaged the integrity of mitochondrial cristae. G,H) Representative image of IF staining for ACAN and MMP3 in in human primary NPCs with or without the treatment of lactate for 24 h, as well as the quantitative results for ACAN and MMP13, respectively (*n* = 3). I) Representative results of H&E and SOFG for mouse IVD tissue treated by needle puncture with or without the administration of lactate (10 × 10^−3^ m, intradiscal injection, 2 µL per mouse). J) Histological score of mouse IVD tissue treated by needle puncture with or without the administration of lactate (10 × 10^−3^ m, intradiscal injection, 2 µL per mouse) (*n* = 5). H&E: Hematoxylin and eosin; SOFG: Safranin O‐Fast Green; NPCs: Nucleus pulposus cells; ECM: Extracellular matrix; MMP3/13: Matrix metalloprotease 1/13; RT‐qPCR: RNA reverse transcription, and quantitative real‐time polymerase chain reaction; IL‐1β: Interleukin 1β; TNFα: Tumor necrosis factor‐α; ACAN: Aggrecan; COL2A1: Type 2 collagen. All data are shown as the mean ± SD. Two‐tailed unpaired Student's *t*‐tests (C, G, I, L, M, and N) and one‐way analysis of variance (ANOVA) were used followed by Tukey's post hoc test (J) to determine the statistical significance. *P < 0.05, **P < 0.01, ***P < 0.001, ****P < 0.0001.

### 2.3 Excessively Increased Lactate Contributes to the Dysfunction and Degeneration of NPCs

Lactate is a major product of glycolysis. Hence, the effects of lactate on NPCs were examined, and cell viability assay suggested that although lactate at very low concentration (0.01 × 10^−3^ m) increased the viability of NPCs. However, lactate from the concentration of 0.1 × 10^−3^ to 20 × 10^−3^ m cause obvious decrease in the cell viability in human NPCs (**Figure** **3**A). Notably, the results of RT‐qPCR indicated that lactate at 10 × 10^−3^ m could result in obvious expression changes of all the IVDD‐related genes, including MMP3, MMP13, IL‐1β, and TNFα (Figure 3B). Collectively, we chose 10 × 10^−3^ m as the experimental concentration of lactate in the following experiments. Further Western blot assay confirmed that lactate at 10 × 10^−3^ m showed promotive effects on ECM degradation in NPCs, including increased expression of MMP3 and MMP13, and decreased expression of ACAN and COL2A1 (Figure 3C,D). As the cellular energy industry, mitochondria play a key role in the regulation of cell function.^[^ ^25^ ^]^ The mitochondrial membrane potential assay revealed that lactate stimulation (10 × 10^−3^ m) decreased the mitochondrial membrane potential of human NPCs in vitro (Figure 3E). IF staining of the mitochondria suggested that the administration of lactate significantly damaged the integrity of the mitochondrial cristae (Figure 3F). IF staining confirmed the pro‐catabolic effect of lactate on the ECM of human NPCs in vitro, as indicated by the expression of ACAN and MMP3 (Figure 3G,H).

For in vivo experiment, we established the IVDD model with and without lactate. Briefly, a sterile needle (attached to a 2‐mL injection syringe) was firstly used to make a perpendicular puncture into the mouse tail IVD. Then, a 5 µL micro‐injection syringe (MICROLITER Series; Hamilton Bonaduz, Switzerland) was used to inject lactate (10 × 10^−3^ m, 2 µL per mouse) into the IVD tissue after the model had been constructed. At the terminal of experiment, and IVD tissues were collected and analyzed. Hematoxylin and eosin (H&E) and safranin O fast green (SOFG) staining showed that the needle resulted in damage to the features of NP tissue, AF tissue, EP tissue, and the boundary among the three parts after four weeks of operation; however, the administration of lactate further aggravated IVDD (Figure 3I,J).

Taken together, the excessive production and accumulation of lactate within the IVD microenvironment could initiate the dysfunction of NPCs and aggravate IVDD.

### 2.4 Integrated Analysis of Bulk RNA Sequencing and Single‐Cell RNA Sequencing Revealed the Activated Effects of Lactate on Ferroptosis in NPCs

**Figure 4:**
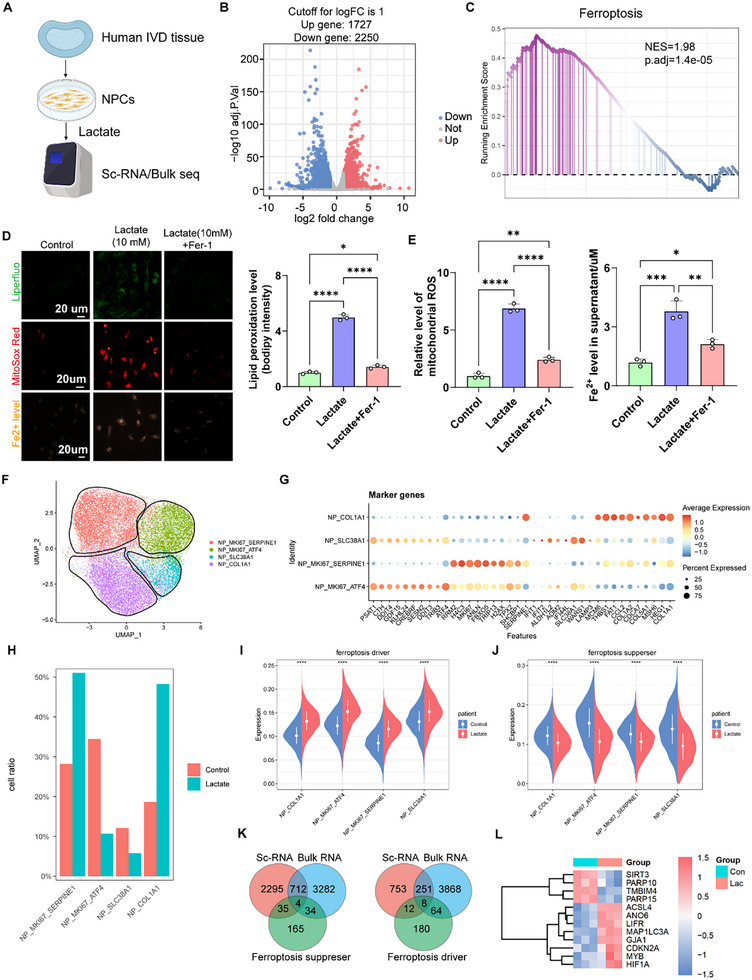
Lactate‐triggered dysfunction of nucleus pulposus is induced by the activation of ferroptosis. A) Illustration of the RNA sequencing process of human primary NPCs treated by lactate (10 × 10^−3^ m) for 24 h. B) The results of Volcano plot indicating all the differentially expressed (upregulated and downregulated) genes in lactate‐treated human primary NPCs (*n* = 3). C) The results of GESA indicating activated ferroptosis signaling pathways in lactate‐treated human primary NPCs. D,E) Representative image of LPO level in lactate‐treated human primary NPCs with or without Ferrostatin‐1 (Fer‐1, a classical ferroptosis inhibitor), as well as the quantitative results for LPO, mitochondrial ROS level and Fe^2+^ level (*n* = 3). F) UMAP visualizing human NPCs as four different cell clusters after unsupervised clustering. Each plot indicated one single cell and colored based on different cell subclusters. G) Representative molecular signatures for each human primary NPC cluster. The area of the bubbles indicates the proportion of cells expressing the gene, and the color intensity reflects the expression intensity. H) Relative proportion of each human primary NPC cluster between control group and lactate group as indicated. I) The expression strength of ferroptosis driver‐related genes between control group and lactate group. J) The expression strength of ferroptosis suppresser‐related genes between control group and lactate group. K) Venn diagram of ferroptosis‐related genes via integrated analysis of bulk RNA sequencing and ScRNA‐seq of human primary NPCs between control group and lactate group. L) Differentially expressed ferroptosis‐related genes via integrated analysis of bulk RNA sequencing andScRNA‐seq between control group and lactate group. LPO: Lipid peroxidation; NPCs: Nucleus pulposus; GSH: Glutathione; NADPH: Nicotinamide adenine dinucleotide phosphate; SOD: Superoxide Dismutase; ROS: Reactive oxygen species; UMAP: Uniform manifold approximation and projection; ScRNA‐seq: Single‐cell RNA sequencing. All data are shown as the mean ± SD. One‐way analysis of variance (ANOVA) was used followed by Tukey's post hoc test (D and E) to determine the statistical significance. *P < 0.05, **P < 0.01, ***P < 0.001, ****P < 0.0001. Scale bar = 20 µm.

To explore the regulatory mechanisms of lactate in NPCs, NPCs were treated with lactate (10 × 10^−3^ m) for 24 h, and the mRNA of the cells was firstly collected for bulk mRNA‐seq (**Figure** **4**A). The volcano plot exhibited the DEGs induced by lactate in human NPCs (Figure 4B). GSEA suggested that ferroptosis was significantly enriched in lactate‐treated NPCs (Figure 4C). In addition, we further confirmed the promotive effect of lactate on ferroptosis using bulk RNA sequencing data of rat nucleus pulposus downloaded from the NCBI's Gene Expression Omnibus (GEO) database (GSE219145) (https://www.ncbi.nlm.nih.gov/geo/query/acc.cgi). (Figure S3A,B, Supporting Information). In vitro experiments demonstrated that lactate administration resulted in increased production of lipid peroxidation (LPO), mitochondrial reactive oxygen species (ROS), and accumulation of Fe^2+^ in human NPCs, which are the three typical characteristics of ferroptosis (Figure 4D,E). However, Ferrostatin‐1 (Fer‐1), an effective and selective ferroptosis inhibitor, suppressed lactate‐induced ferroptosis‐related phenotypes, as evidenced by the results of LPO, mitochondrial ROS, and Fe^2+^ detection assays (Figure 4D,E). The presence of polyunsaturated fatty acids (PUFA) at the bis‐allylic position of phospholipids is critical for phospholipid peroxidation and ferroptosis. Therefore, a PUFA‐enriched NPC model was created to investigate the role of lactate on ferroptosis susceptibility.^[^ ^26^ ^]^ NPCs were incubated with linoleic acid or oleic acids for 24 h together with lactate (Figure S4A, Supporting Information). The results showed that pre‐treatment with lactate and PUFA decreased NPC survival and the levels of glutathione (GSH) and nicotinamide adenine dinucleotide phosphate (NADPH) (Figure S4B, Supporting Information). Therefore, the above results show that excessive accumulation of lactate increases the susceptibility of NPCs to ferroptosis.

To identify the critical molecular players of lactate in the regulation of ferroptosis activation, we performed single‐cell RNA sequencing (ScRNA‐seq) of human NPCs with and without lactate treatment (Figure 4A). UMAP analysis primarily classified human NPCs into four different clusters: MKI67 (Marker of proliferative protein Ki‐67)^+^SERPINE1 (Serpin family E member 1)^+^ NPCs, MKI67^+^ATF4 (Activating transcription factor 4)^+^ NPCs, SLC38A1 (Solute carrier family 38 member 1)^+^ NPCs, and COL1A1 (Type 1 collagen)^+^ NPCs (Figure 4F). Dot plots of marker gene expression are shown for the NPC subtypes (Figure 4G). The proportion analysis of NPC clusters in different groups showed that the proportion of MKI67^+^SERPINE1^+^ and COL1A1^+^ NPCs increased, but that of MKI67^+^ATF4^+^ and SLC38A1^+^ NPCs decreased in lactate‐induced NPCs (Figure 4H). Next, we evaluated the effect of lactate on pro‐ and anti‐ferroptosis‐related gene expression in NPC clusters and found that ferroptosis drivers were all activated, whereas ferroptosis suppressors were inhibited, further validating that lactate activates ferroptosis in NPCs (Figure 4I,J). We then integrated the data from bulk and single‐cell RNA sequencing and discovered that the expression of four ferroptosis suppressors, including SIRT3, poly(ADP‐ribose) polymerase family member 10 (PARP10), Transmembrane Bax inhibitor‐1 motif‐containing 4 (TMBIM4), and PARP15, were repressed by lactate, and that the expression of eight ferroptosis drivers, including Acyl‐CoA synthetase long‐chain family (ACSL4), Anoctamin 6 (ANO6), leukemia inhibitory factor receptor (LIFR), microtubule‐associated protein 1 light chain 3 alpha (MAP1LC3A), gap junction protein alpha1 (GJA1), cyclin‐dependent kinase inhibitor 2 (CDKN2A), myeloblastosis (MYB), and HIF‐1α was enhanced (Figure 4K,L). However, blocking the uptake of lactate into human NPCs significantly inhibited the expression of ferroptosis drivers and alleviated the expression of ferroptosis suppressors (Figure S5A–D, Supporting Information).

Collectively, these results suggest that abnormal lactate production can activate ferroptosis in NPCs by regulating the expression of pro‐ and anti‐ferroptosis‐related genes.

### 2.5 Lactate Amplified Phospholipid Peroxidation to Induce Ferroptosis via promoting H3K18la‐mediated gene transcription of *ACSL4*

**Figure 5:**
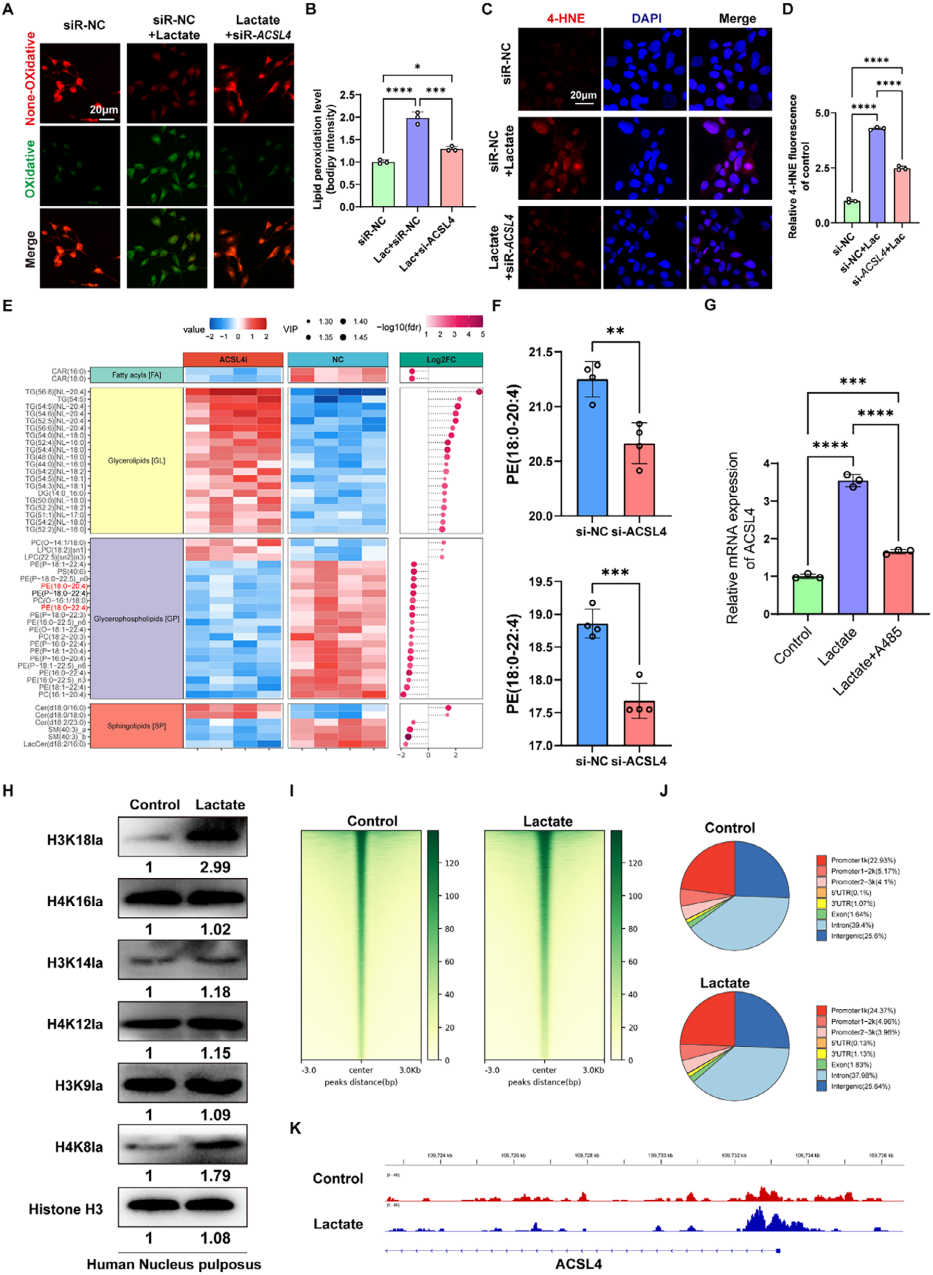
Lactate amplified phospholipid peroxidation to induce ferroptosis via promoting H3K18la‐mediated gene transcription of *ACSL4*. A,B) Representative image of LPO level in lactate‐treated human primary NPCs with or without *ACSL4* silencing, as well as the quantitative results for LPO (*n* = 3). C,D) Representative image of IF staining for 4‐HNE in lactate‐treated human NPCs with or without *ACSL4* silencing, as well as the quantitative results for LPO (*n* = 3). E) Heat map showed the differentially produced lipids in lactate‐enriched human primary NPCs with or without silencing ACSL4. Each lipid species was normalized to the corresponding mean value. F) The relative level of PE (C18:0‐C20:4) and PE (C18:0‐C22:4), which have been reported to be the most vulnerable phospholipids to peroxidation (*n* = 4). G) RT‐qPCR results of ACSL4 expression in lactate‐treated human primary NPCs with or without A485 (5 × 10^−6^ m), a highly selective catalytic p300/CBP inhibitor, for 24 h (*n* = 3). H) Western blotting analysis of site‐specific histone lactylation in the control and lactate‐treated human primary NPCs. I) The binding density of H3K18la was visualized by deepTools: the heatmap presents the CUT&Tag tag counts on the different H3K18la binding peaks in control and lactate‐treated human primary NPCs, ordered by signal strength. J) The distribution of H3K18la on the genome in control and lactate‐treated human primary NPCs. K) Genome browser tracks of CUT&Tag signal at the representative target gene loci indicated enriched H3K18la in the promotors of ACSL4. ACSL4: Acyl‐CoA Synthetase Long Chain Family Member 4; RT‐qPCR: RNA reverse transcription, and quantitative real‐time polymerase chain reaction; LPO: Lipid peroxidation; TEM: Transmission electron microscopy; NPCs: Nucleus pulposus. All data are shown as the mean ± SD. Two‐tailed unpaired Student's *t*‐tests (F) and one‐way analysis of variance (ANOVA) were used followed by Tukey's post hoc test (B, D, and G) to determine the statistical significance. *P < 0.05, **P < 0.01, ***P < 0.001, ****P < 0.0001.

Phospholipid peroxidation initiates ferroptosis (Figure S6A, Supporting Information).^[^ ^27^ ^]^ Among the DEGs induced by lactate, the acyl‐CoA synthetase long‐chain family (ACSL4), a critical promoter of phospholipid peroxidation, was significantly upregulated (Figure S6A, Supporting Information). The in vitro assays showed that lactate dose‐dependently upregulated the mRNA expression of ACSL4 (Figure S6B, Supporting Information). Subsequently, we evaluated the LPO level in NPCs, and the BODIPY assay indicated that silencing *ACSL4* significantly restored LPO in lactate‐induced human NPCs (**Figure** **5**A,B). 4‐Hydroxy‐2‐nonenal (4‐HNE) is also a well‐known by‐product of lipid peroxidation and an important marker of ferroptosis;^[^ ^28^ ^]^ therefore, we examined 4‐HNE accumulation in NPCs. The results showed that lactate increased 4‐HNE levels, whereas inhibition of *ACSL4* expression evidently ameliorated this effect (Figure 5C,D). Transmission electron microscopy (TEM) further revealed that lactate pretreatment resulted in a significant decrease in the mitochondrial cristae of NPCs, whereas the number of ferroptosis‐related mitochondria decreased after silencing ACSL4 (Figure S6C,D, Supporting Information). Notably, we explored the effect of lactate on the susceptibility of human NPCs to ferroptosis. In a PUFA‐enriched environment, lactate‐mediated ferroptosis was suppressed by ACSL4 knockdown, as indicated by the results of LPO, 4‐HNE accumulation, and mitochondrial morphology (Figure S7, Supporting Information). Moreover, we performed lipidomic analysis of lactate‐treated NPCs with and without silencing of ACSL4 (Figure 5E). Among the lipids, PE (C18:0–C20:4) and PE (C18:0–C22:4) have been reported to be the most vulnerable to peroxidation, which was significantly inhibited after silencing ACSL4.^[^ ^29^ ^]^ The results of differentially abundant lipids showed that inhibiting the expression of ACSL4 resulted in lipidomic changes in different long‐chain PUFA‐containing phospholipid species in lactate‐induced NPCs, including 147 downregulated and 91 upregulated lipids (Figure 5F). Therefore, lactate amplifies phospholipid peroxidation to induce ferroptosis by increasing the gene transcription of ACSL4.

To investigate whether lactate regulated the transcription of ACSL4 via histone lactylation, we firstly examined the effect of lactylation on ACSL4 expression. Given that p300/CBP being the major writer for lactylation, we chose A485 (5 × 10^−6^ m), a highly selective catalytic p300/CBP inhibitor, to treated human NPCs, and the results showed that inhibiting p300/CBP‐mediated lactylation significantly decreased ACSL4 expression, suggesting lactylation may be involved in ACSL4 transcription (Figure 5G). As previous studies reported, the promotive effects of lactate on gene expression depended mainly on histone lactylation.^[^ ^30^, ^31^ ^]^ We then examined the expression of lactylation histone. After the NPCs were treated with lactate (10 × 10^−3^ m) for 48 h, we collected the protein for subsequent western blot assay. The results showed that lactate increased the lactylation level of the most histone, including H3K18la, H4K12la, and H4K8la, of which the increase in H3K18 lactylation is the most significant (Figure 5H). Then, we further examined the relationship between H3K18la and ACSL4, we performed the genome‐wide CUT&TAG analysis to identify the transcriptional regulation of ACSL4 by H3K18la in human NPCs with or without lactate. Briefly, total protein was collected by magnetic separation: CUT&TAG using antibodies against H3K18la and analysis with deepTools3 revealed obvious enrichment of H3K18la peaks in lactate‐treated nucleus pulposus compared with the control group (Figure 5I). The enrichment of H3K18la in promoter regions and upstream regions of genes under lactate stimulus was elevated from 32.17% to 33.29% (Figure 5J). Specifically, the called peaks identified candidate genomic loci showed that the levels of H3K18la at promoters of ACSL4 were elevated (Figure 5K). Combined the results from RT‐qPCR above, we could conclude that lactate increased the transcription level of ACSL4 via elevated H3K18la level.

### 2.6 Lactate‐Induced ACSL4 Lactylation to Enhance Ferroptosis in NPCs

Post‐translational modifications (PTMs) play a significant role in regulating the function of proteins and cell phenotypes in response to microenvironmental variations.^[^ ^32^ ^]^ A recent study identified a novel PTM induced by lactate called lysine lactylation that regulates protein function.^[^ ^32^ ^]^ Although we have shown that lactate promotes the transcription of *ACSL4* via H3K18la; however, whether lactate regulates ACSL4 function via lactylation remains largely unknown.

**Figure 6:**
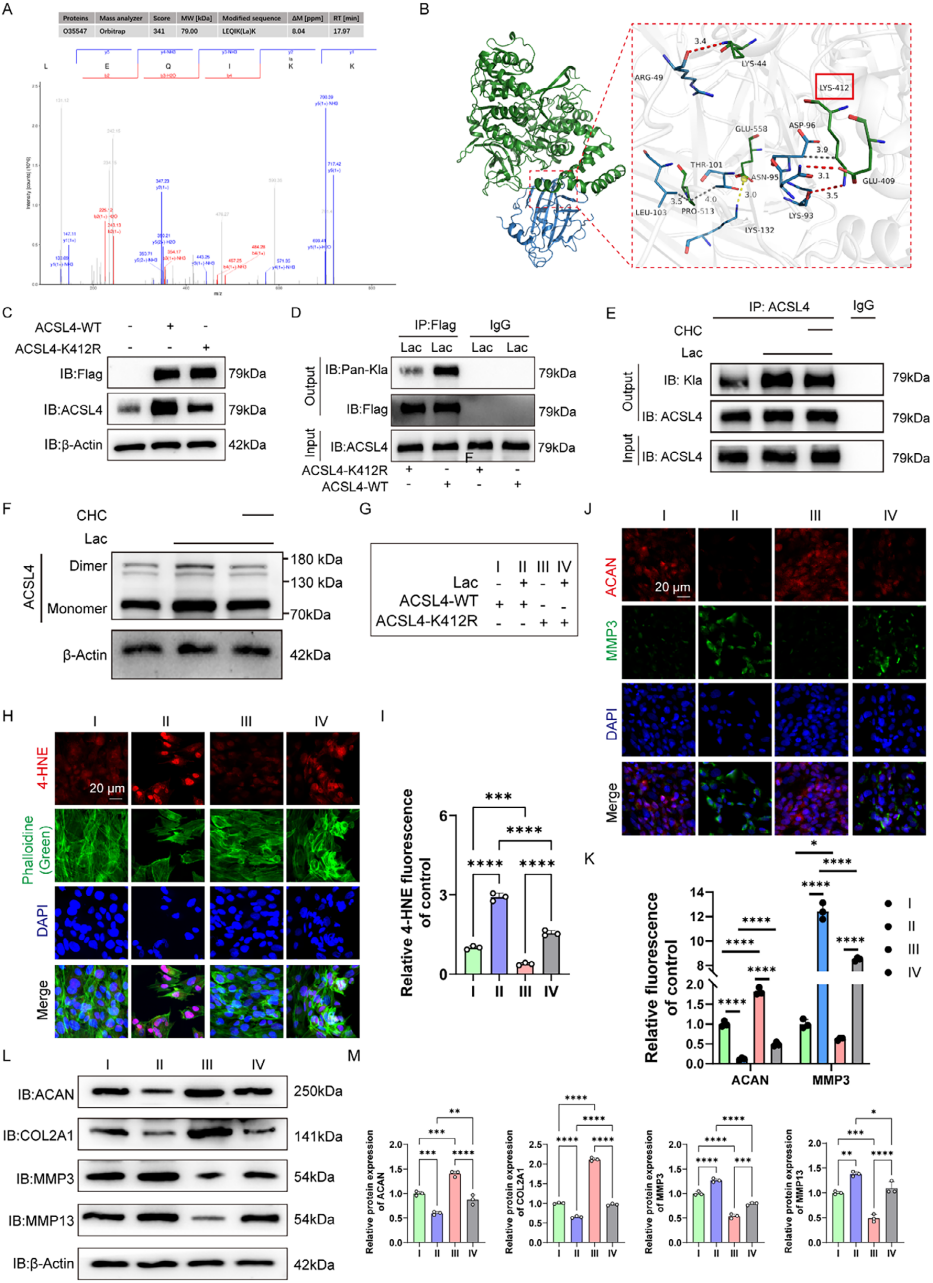
Lactate‐induced ACSL4 lactylation to enhance ferroptosis in NPCs. A) Mass spectra for ACSL4 peptides lactylated at K412 in human primary NPCs. B) Ribbon diagram of the crystal structure of human ACSL4 protein and lactylation group. C) K412R site mutation and wild‐type flag‐ACSL4 overexpressed 293T cells were constructed, respectively, through overexpression plasmid. Cell proteins were analyzed by Western blotting (WB) for ACSL4 levels. D) Flag‐ACSL4 overexpressed human primary NPC proteins were pulled down by ACSL4 antibody and detected with anti‐lactyllysine antibody. E) Representative blot of Western blot for the protein level of Kla and ACSL4 in human primary NPCs treated with the MCT inhibitor CHC before lactate administration. F) Representative blot of Western blot for the protein level of ACSL4 monomer and dimer in human primary NPCs treated with the MCT inhibitor CHC before lactate administration. G) Illustration of the cell groups; H,I) representative image of IF staining for 4‐HNE and phalloidine in K412R site mutation and wild‐type flag‐ACSL4 overexpressed NPCs in the presence of lactate, as well as the quantitative results for LPO (*n* = 3). J,K) Representative image of IF staining for MMP3 and ACAN in K412R site mutation and wild‐type flag‐ACSL4 overexpressed NPCs in the presence of lactate, as well as the quantitative results (*n* = 3). L,M) Representative blot of Western blot for the protein level of ACAN, COL2A1, MMP3, MMP13, and β‐ACTIN in K412R site mutation and wild‐type flag‐ACSL4 overexpressed NPCs in the presence of lactate, as well as the quantitative results (*n* = 3). ACSL4: Acyl‐CoA Synthetase Long Chain Family Member 4; NPCs: Nucleus pulposus cells; GAPDH: Glyceraldehyde‐3‐phosphate dehydrogenase; 4‐HNE: 4‐hydroxy‐2‐nonenal; LPO: Lipid peroxidation; ACAN: Aggrecan; COL2A1: Type 2 collagen; MMP3/13: Matrix metalloprotease 1/13. All data are shown as the mean ± SD. One‐way analysis of variance (ANOVA) was used followed by Tukey's post hoc test (I, K, and M) to determine the statistical significance. *P < 0.05, **P < 0.01, ***P < 0.001, ****P < 0.0001.

We firstly used an ACSL4 antibody to pull down ACSL4 and an anti‐lactyllysine antibody to determine the lactylation site of ACSL4 in lactate‐induced human NPCs. A significant increase in the MS peak intensity was observed at the K412 site of ACSL4 after lactate administration, indicating that this site may be the lactylation site of ACSL4 (**Figure** **6**A). As seen in the ACSL4 tertiary structure, K412 is located within the functional domain of the ACSL4 protein (Figure 6B). Notably, the K412 site is relatively conserved in multiple mammals (Figure S8, Supporting Information). We then constructed a flag‐tagged K412R site mutation and WT ACSL4 via an overexpression plasmid into HEK 293 T cells. Compared to the control group, the protein expression of mutant or WT ACSL4 in 293T cells was significantly upregulated (Figure 6C). Subsequently, we utilized the FLAG antibody to pull down ACSL4, and evaluated the effect of K412 site on lactylation levels in human NPCs treated with lactate. Compared to WT ACSL4‐overexpressing NPCs, NPCs overexpressing mutant ACSL4 showed significantly lower ACSL4 lactylation levels (Figure 6D). Furthermore, inhibition of the transmembrane transport of lactate by CHC administration alleviated lactate‐induced lactylation of ACSL4, suggesting the promotive effects of supplementary lactate on ACSL4 lactylation (Figure 6E). Dimerization of ACSL4 is essential for its enzyme activation. Therefore, we evaluated whether the lactylation of ACSL4 increases the formation of ACSL4 dimer. As shown in the results, the amount of ACSL4 dimer also in‐creased in response to lactate, but decreased in response to α‐cyano‐4‐hydroxycinnamic acid (CHC) (Figure 6F).

Furthermore, we evaluated the effect of K412 lactylation on ferroptosis in human NPCs. The intracellular contents of GSH and NADPH in NPCs with the ACSL4 with mutated K412 site were higher than those in human NPCs with WT ACSL4 after lactate administration (Figure S9, Supporting Information). We also found that lactate treatment markedly promoted 4‐HNE accumulation in human NPCs with WT ACSL4, whereas NPCs with the K412 site mutation in ACSL4 showed reduced production of 4‐HNE with and without lactate administration, indicating that the K412 site mutation of ACSL4 partially alleviated lactate‐induced ferroptosis (Figure 6G–I). ECM metabolism was also evaluated. IF staining for ACAN and MMP3 suggested a tendency consistent with the western blot results (Figure 6J,K). Western blotting showed that in NPCs with the WT ACSL4 or K412 site mutation of ACSL4, lactate decreased the expression of ACAN and COL2A1, but increased the expression of MMP3 and MMP13. However, NPCs with the ACSL4 with K412 site mutation exhibited less ECM imbalance (Figure 6L,M).

Collectively, these results indicate that in addition to increasing the mRNA expression of *ACSL4*, lactate can enhance the activity of ACSL4 via lactylation and that the K412 site is a potential regulatory site for the function of ACSL4.

### 2.7 Lactate‐mi‐RNA Axis Mediated Downregulation of SIRT3 Was Involved in ACSL4 Lactylation

**Figure 7:**
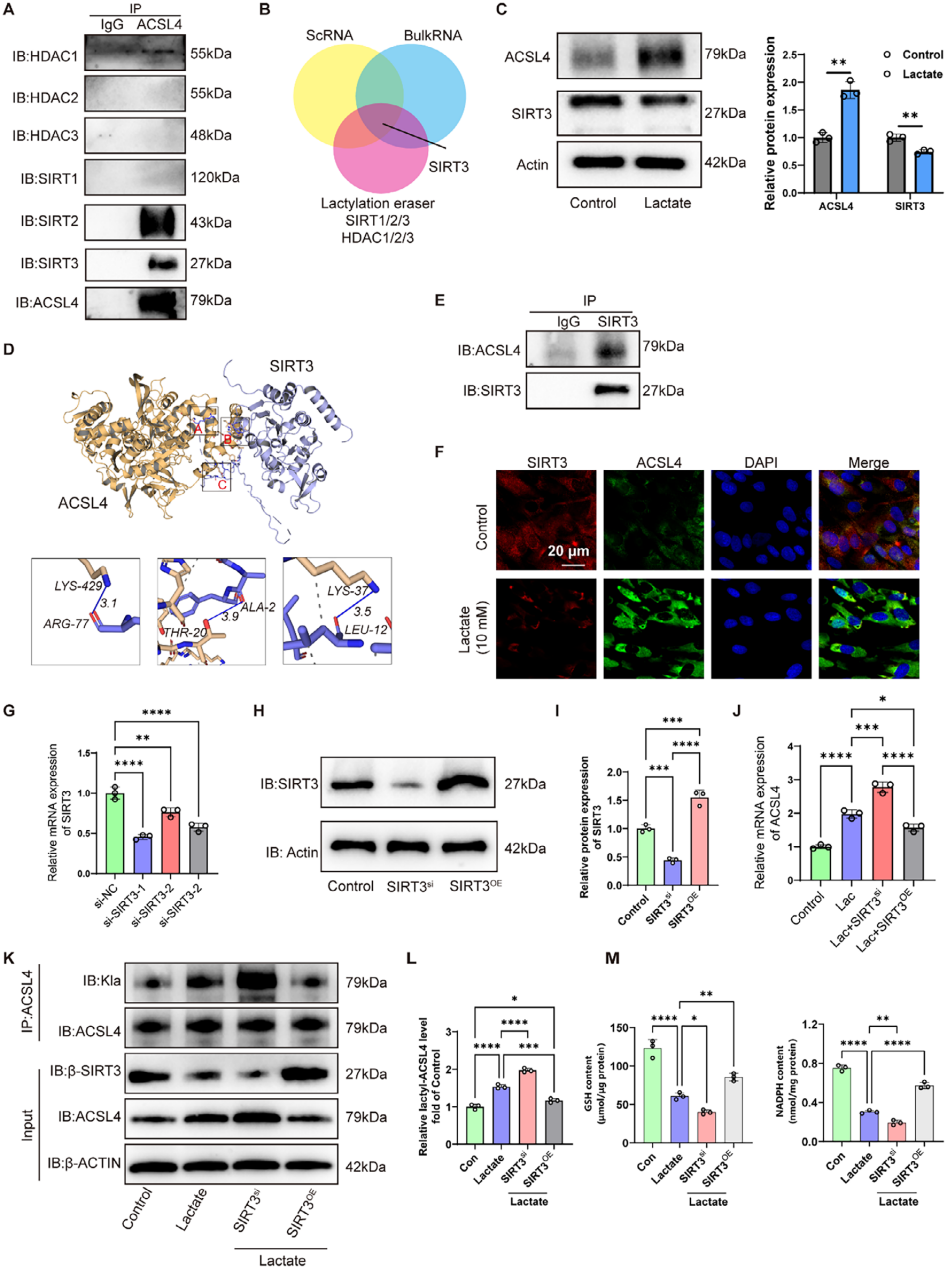
SIRT3 was the potential eraser to regulate ACSL4 lactylation. A) Co‐immunoprecipitation (co‐IP) assay between these erasers (sirtuin 1–3 and histone deacetylase 1–3) and ACSL4. B) Integrated analysis lactylation‐related genes based on bulk and ScRNA‐seq and potential erasers. C) Representative blot of Western blot for the protein expression of ACSL4 and SIRT3 in human primary NPCs with or without lactate (10 × 10^−3^ m), as well as the quantitative results (*n* = 3). D) Molecular docking diagram of ACSL4 and SIRT3. E) Reverse Co‐IP revealed that ACSL4 was precipitated by SIRT3 in NPCs. F) Representative images of IF staining of the co‐location between SIRT3 and ACSL4 in human NPCs with or without lactate. G) RT‐qPCR assay to detect the best siRNA for SIRT3 silencing (*n* = 3). H,I) Representative blot of Western blot for the protein expression of SIRT3 in SIRT3‐silencing or overexpressing human primary NPCs as well as the quantitative results (*n* = 3). J) RT‐qPCR assay to detect the effect of SIRT3 silencing or overexpression on the mRNA level of ACSL4 (*n* = 3). K,L) Representative blot of Western blot for the protein level of Kla and ACSL4 in SIRT3‐silencing or overexpressing human primary NPCs, as well as the quantitative results (*n* = 3). M) Intracellular level of GSH and NADPH in SIRT3‐silencing or overexpressing human NPCs (*n* = 3). ScRNA‐seq: Single‐cell RNA sequencing; ACSL4: Acyl‐CoA Synthetase Long Chain Family Member 4; NPCs: Nucleus pulposus cells; SIRT3: Sirtuin‐3; GSH: Glutathione; NADPH: Nicotinamide adenine dinucleotide phosphate. All data are shown as the mean ± SD. Two‐tailed unpaired Student's *t*‐tests (C and G) and one‐way analysis of variance (ANOVA) were used followed by Tukey's post hoc test (I, J, L, and M) to determine the statistical significance. *P < 0.05, **P < 0.01, ***P < 0.001, ****P < 0.0001.

In addition to the writer‐mediated protein lactylation, lactylation can also be regulated by eraser proteins include sirtuin 1–3 (SIRT1–3) and histone deacetylase 1–3 (HDAC1–3).^[^ ^33^ ^]^ To further identify the critical eraser for ACSL4, we performed the co‐immunoprecipitation (co‐IP) assay between these erasers and ACSL4. Among the studied enzymes, the results showed the evident interactions between ACSL4 and HDAC1, SIRT2, and SIRT3 (**Figure** **7**A). Through an integrated analysis of bulk RNA and scRNA‐seq, we found that SIRT3 was the only erasers that can be regulated by lactate (Figure 7B). Furthermore, Western blot confirmed that lactate resulted in clear increased expression of ACSL4 and decreased expression of the SIRT3 (Figure 7C). Thereafter, we performed a molecular docking analysis (http://hdock.phys.hust.edu.cn/), and the results revealed that ACSL4 could well bind to SIRT3 through the formation of three hydrogen bonds between ARG‐77 and LYS‐429, ALA‐2 and THR‐20, and LEU‐12 and LYS‐37 (Figure 7D). In addition, reverse co‐IP in human NPCs further demonstrated that ACSL4 was significantly precipitated by SIRT3 (Figure 7E). Co‐localization analysis using IF staining further validated the interaction in human NPCs with or without lactate (Figure 7F).

Furthermore, we selected the best sequence for SIRT3 silencing (Figure 7G). Then, we knocked down and overexpressed SIRT3 in NPCs using siRNA and overexpression plasmids, which was confirmed by Western blot assays (Figure 7H,I). Notably, we evaluated the effect of SIRT3 silencing on ACSL4 expression induced by lactate, and found that SIRT3 overexpression did not completely reversed lactate‐induced ACSL4 transcription, which suggested that increased expression and of ACSL4 by lactate is not SIRT3‐dependent (Figure 7J). Furthermore, we evaluated the level of ACSL4 expression and lactylation in human NPCs with SIRT3 knockdown and overexpression in the presence of lactate. The results demonstrated that inhibiting SIRT3 expression could further increase lactate‐induced ACSL4 expression and lactylation, whereas enhancing SIRT3 expression significantly alleviated these effects (Figure 7K,L). In addition, we determined the intracellular levels of GSH and NADPH to evaluate ferroptosis, and found that downregulation of SIRT3 expression further decreased the levels of GSH and NADPH in lactate‐induced NPCs, whereas overexpression of SIRT3 significantly restored the intracellular levels of GSH and NADPH (Figure 7M). Collectively, we deduced that SIRT3 was the potential eraser to regulate ACSL4 lactylation in human NPCs.

In addition, we also investigated the potential mechanism of lactate in regulating SIRT3 expression. miRNAs with the length of 22–25 nucleotides are the major group of small non‐coding RNAs, which participate in the development of many diseases including intervertebral disc degeneration via directly inhibiting the mRNA levels for target genes.^[^ ^34^ ^]^ Therefore, we first sought the potential bridge miRNAs that targeting SIRT3 using the algorithm Target Scan from Target Scan Human 7.2, mirDIP, and miRDB. The database cross‐check identified 21 potential miRNAs that targeted SIRT3 mRNA 3′‐UTR (Figure S10A, Supporting Information). Then, we divided human NPCs into control group and lactate‐treated group and performed the RT‐qPCR analysis. The results showed that only miR‐708‐5p was significantly increased by lactate (Figure S10B, Supporting Information). In addition, the luciferase reporter assay was carried out and the results showed that SIRT3 expression could be directly modulated by miR‐708‐5p via interactions with potential binding sites in lactate‐induced NPCs. As show in Figure S10C (Supporting Information), perfect base paring was found between the seed sequence of miR‐708‐5p and the 3′UTR of SIRT3 (Figure S10C, Supporting Information). The results showed that human NPCs co‐transfected with miR mimic and WT SIRT3 presented a significant decrease in luciferase activity in comparison with miR‐NC group (Figure S10D, Supporting Information). There was no obvious change in the luciferase activity in MUT SIRT3 group either transfected with miR‐708‐5p mimic or mimic‐NC (Figure S10D, Supporting Information). In conclusion, miR‐708‐5p can be a potential bridge for lactate in regulating SIRT3.

### 2.8 Inhibiting Glycolysis‐Derived Lactate Suppressed Ferroptosis Activation and Ameliorated IVDD In Vivo

**Figure 8:**
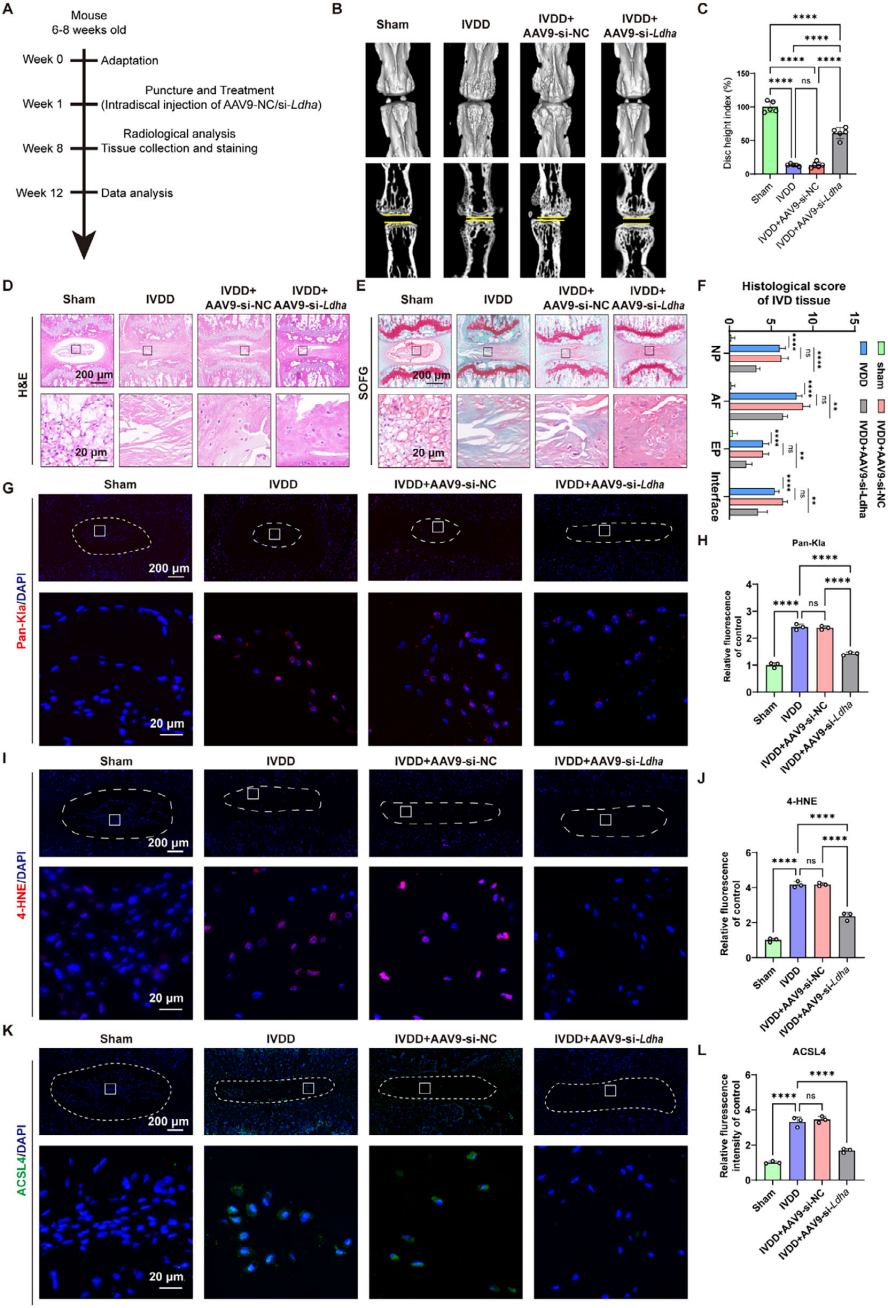
Inhibiting glycolysis ameliorates protein lactylation and ACSL4‐induced ferroptosis and IVDD in mice. A) Illustration of the animal experiment design to investigate the effect of decreasing lactate content via silencing *Ldha* on IVDD in vivo. B) Representative images of micro‐CT in evaluating the disc height of mouse tail IVD in sham, IVDD, IVDD+AAV9‐siNC, and IVDD+AAV9‐siLdha group, respectively. C) Disc height index of mouse IVD in sham, IVDD, IVDD+AAV9‐siNC, and IVDD+AAV9‐siLdha group, respectively (*n* = 5). D) Representative images of H&E staining of mice tail IVD tissue in sham, IVDD, IVDD+AAV9‐siNC, and IVDD+AAV9‐siLdha group, respectively. E) Representative images of SOFG staining of mice tail IVD tissue in sham, IVDD, IVDD+AAV9‐siNC, and IVDD+AAV9‐siLdha group, respectively. F) Histological score of mice tail IVD tissue in sham, IVDD, IVDD+AAV9‐siNC, and IVDD+AAV9‐siLdha group, respectively (*n* = 5). G,H) Representative images and quantitative results of IF staining for Pan‐Kla of mice tail IVD tissue in sham, IVDD, IVDD+AAV9‐siNC, and IVDD+AAV9‐siLdha group, respectively (*n* = 3). I,J) Representative images and quantitative results of IF staining for 4‐HNE of mice tail IVD tissue in sham, IVDD, IVDD+AAV9‐siNC, and IVDD+AAV9‐siLdha group, respectively (*n* = 3). K,L) Representative images and quantitative results of IF staining for ACSL4 of mice tail IVD tissue in sham, IVDD, IVDD+AAV9‐siNC, and IVDD+AAV9‐siLdha group, respectively (*n* = 3). ACSL4: Acyl‐CoA Synthetase Long Chain Family Member 4; LDHA: Lactate dehydrogenase A; IVDD: Intervertebral disc degeneration; SOFG: Safranin O‐Fast Green; H&E: Hematoxylin and eosin; AAV9: Adenoviral‐associated viruses 9; 4‐HNE: 4‐hydroxy‐2‐nonenal. All data are shown as the mean ± SD. One‐way analysis of variance (ANOVA) was used followed by Tukey's post hoc test (C, F, H, J, and L) to determine the statistical significance. *P < 0.05, **P < 0.01, ***P < 0.001, ****P < 0.0001.

Delaying the process of IVDD is the ultimate goal of current therapy. Given the promoting effects of glycolysis‐derived lactate on NPC degeneration, we investigated the potential therapeutic effects of glycolysis inhibition on IVDD. Therefore, we established a mouse tail needle model or sham surgery, followed by the intradiscal administration of Adenoviral‐associated viruses 9 (AAV9)‐si*Ldha* (**Figure** **8**A). The IVD samples were collected at the end of the experiment, and IVDD was evaluated by micro‐ (micro‐CT), H&E, Safranin‐O, and IF staining (Figure 8A). Disc height analyses based on micro‐CT showed that the disc height index of mice notably decreased in the IVDD group and IVDD+AAV9‐si‐NC group compared to the WT group, whereas the disc height index increased in the IVDD+AAV9‐si‐*Ldha* group compared to IVDD group and IVDD+AAV9‐si‐NC group (Figure 8B,C).

H&E and SOFG staining revealed that the needle led to reduced numbers of NPCs and proteoglycan content, accompanied by disorganized annulus fibrosus (AF) lamellae and a vanishing boundary between the NP and AF tissue in the IVDD group and IVDD+AAV9‐si‐NC group (Figure 8D–F). Unsurprisingly, AAV9‐si*Ldha* treatment successfully ameliorated the histological damages, including the subfeatures of NP tissue, AF tissue, EP tissue, and the interface among the three parts, induced by the needle, and the histological scores of mice in IVDD+AAV9‐si‐*Ldha* group were higher than those of IVDD group and IVDD+AAV9‐si‐NC group (Figure 8D–F). IF staining using an anti‐lactyl lysine antibody in NP tissue revealed that inhibiting the expression of *Ldha* significantly suppressed needle‐induced lactylation within NP tissue (Figure 8G,H). Next, we evaluated ferroptosis in vivo, and found that needle surgery increased the accumulation of 4‐HNE, a classical biomarker of lipid peroxidation, indicating the activation of ferroptosis in IVDD model (Figure 8I,J). However, silencing *Ldha* via AAV9 dramatically attenuated the production of 4‐HNE (Figure 8I,J). In addition, we also proved that needle induced the expression of ACSL4 within NP tissue, whereas treatment with AAV9‐ AAV9‐si‐*Ldha* significantly suppressed its expression (Figure 8K,L). ECM metabolism analysis suggested that knocking down *Ldha* restored the expression of ACAN, consistent with the results of SOFG staining (Figure S11A,B, Supporting Information). These results above demonstrate the therapeutic effect of suppressing lactate production via gene modulation in IVDD in vivo.

2‐Deoxy‐d‐glucose (2‐DG) has been widely used to inhibit glycolysis.^[^ ^35^ ^]^ As a result, we further explored the treating potential of 2‐DG administration on IVDD. Cell viability assay showed that the concentration of 2‐DG between 0 and 100 × 10^−6^ m showed no cytotoxic effects on NPC proliferation, and 2‐DG at the concentration of 1 × 10^−6^ m showed the best promotive effects on NPC proliferation (Figure S12A, Supporting Information). Therefore, we chose 2‐DG at this dose (1 × 10^−6^ m) for the following in vitro and in vivo experiments. As the results shown, 2‐DG significantly suppressed IL‐1β‐induced lactate secretion (Figure S12B, Supporting Information). Subsequently, gene expression analysis demonstrated that pre‐treated NPCs with 2‐DG (1 × 10^−6^ m) could effectively ameliorate IL‐1β‐induced ECM metabolism disequilibrium, including promoting the expression of ACAN suppressing the expression of MMP3 (Figure S12C, Supporting Information). Consistently, the mouse tail IVDD model was established using needle surgery, followed by intraperitoneal administration of 2‐DG (0.5 g kg^−1^, twice a week for the first two weeks) (Figure S12D, Supporting Information). Radiological analyses showed that treatment with 2‐DG evidently restored the disc height of mouse IVD compared to IVDD+PBS group (Figure S12E,F, Supporting Information). Histological analysis uncovered the indistinct boundaries between NP and AF tissues and decreased glycosaminoglycan (GAG) in the IVDD group (Figure S12G,H, Supporting Information). Nevertheless, these degenerative changes, including the features of NP tissue, AF tissue, EP tissue, and the interface among the three parts, were effectively alleviated by 2‐DG (Figure S12G–I, Supporting Information). In addition, IF staining of IVD tissue demonstrated that, in comparison to the IVDD+PBS group, 2‐DG inhibited the expression of ACSL4 and increased the expression of ACAN (Figure S12J–L, Supporting Information).

Collectively, inhibiting glycolysis through gene silencing or chemical treatment could suppress lactate production and lactylation, thereby significantly ameliorating ferroptosis activation and NPC dysfunction during IVDD.

## 3 Discussion

**Figure 9:**
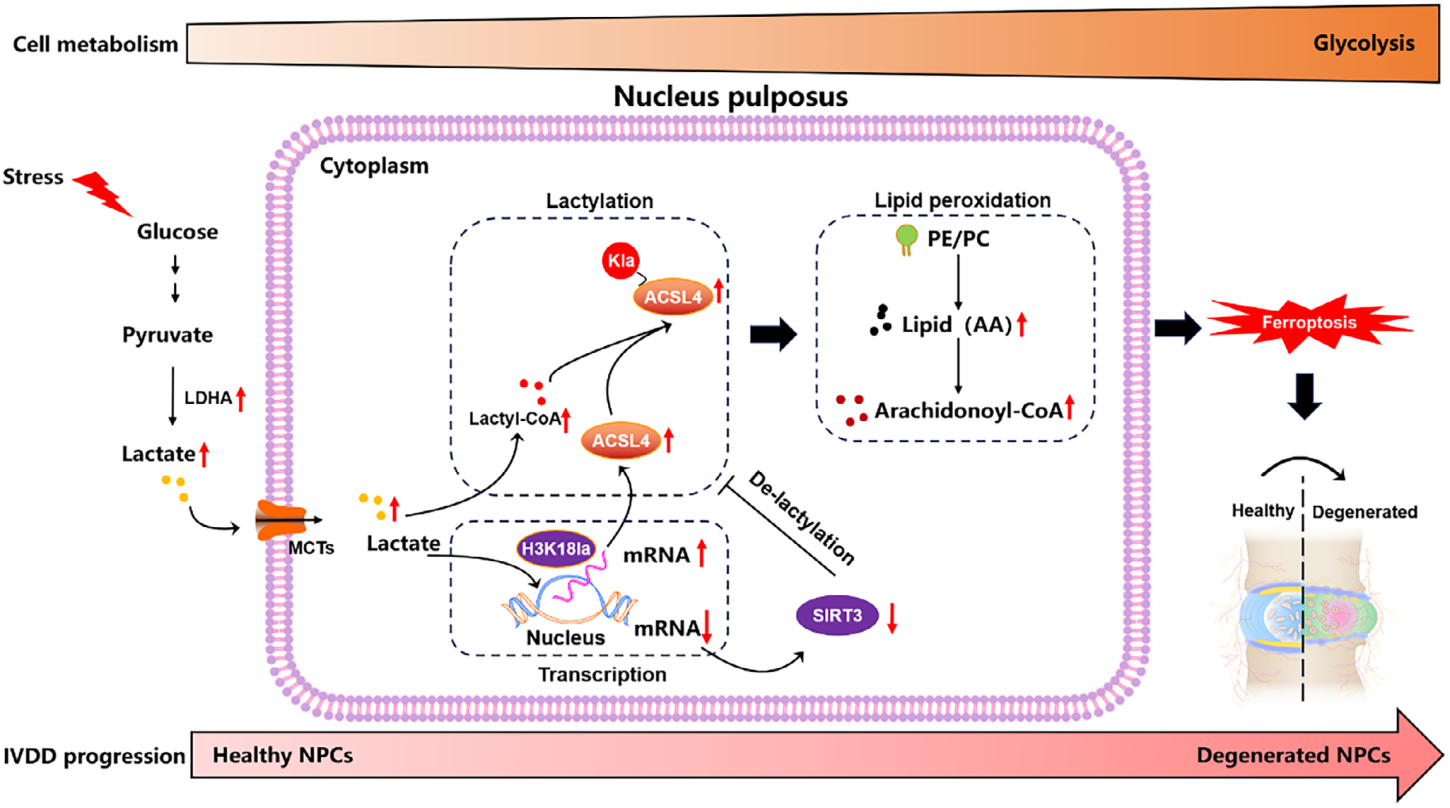
Molecular mechanisms of glycolysis‐derived lactate‐induced ACSL4 expression and lactylation to activate ferroptosis during IVDD. 1) During the process of IVDD, NPCs underwent metabolic remodeling, characterized by excessively increased glucose glycolysis and decreased oxidative phosphorylation, 2) lactate‐triggered NPC dysfunction and IVDD was mediated by the activation of ferroptosis, 3) lactate promoted the gene transcription of ACSL4 via elevated H3K18la level, 4) lactate promoted the lactylation of ACSL4 to enhance ferroptosis, and lactate‐induced decreased expression of SIRT3 further resulted in the elevation of ACSL4 lactylation. ACSL4: Acyl‐CoA Synthetase Long Chain Family Member 4; SIRT3: Sirtuin‐3; IVDD: Intervertebral disc degeneration.

In the present study, we comprehensively explored the glucose metabolism, in NPCs with different degeneration grades based on ScRNA‐seq data, and revealed a previously unknown role of lactate in IVDD by activating ferroptosis. This study highlighted the relationship between metabolic reprogramming and IVDD. In this study, we demonstrated that: 1) NPCs were characterized by a significant increase in glycolysis and a decrease in oxidative phosphorylation during IVDD; 2) lactate resulted in NPC ferroptosis and dysfunction by promoting H3K18‐mediated ACSL4 transcription and lactylation; and 3) suppressing glycolysis through gene silencing or chemical means decreased NPC lactylation level and ferroptosis to alleviate IVDD (**Figure** **9**).

Glycolysis and tricarboxylic acid cycle (TAC) are the two primary pathways that generate ATP.^[^ ^36^ ^]^ Plasticity is a critical characteristic of metabolic processes, and a lack of nutrients or oxygen supply has been widely considered a driver of metabolic reprogramming. In addition to ATP production, metabolic remodeling is closely correlated with cellular dysfunction, including the tumorigenesis, activation of immune cells, regulation of mitochondrial dysfunction, and ECM modulation.^[^ ^37^, ^38^, ^39^ ^]^ In chondrocytes of patients with osteoarthritis (OA), anaerobic glycolysis is enhanced and mitochondrial function is decreased.^[^ ^15^ ^]^ Li et al.^[^ ^17^ ^]^ reported that programmed activation of GLUT1 could result in the accumulation of AGEs and ECM degradation in articular cartilage. Despite the similarities between OA and IVDD, studies on the relationship between metabolic remodeling and IVDD are limited. Li et al.^[^ ^40^ ^]^ found that inhibiting glycolysis could alleviate compression‐induced NPC apoptosis. Wu et al. revealed that lactate downregulated ECM synthesis and promoted apoptosis in rat NPCs.^[^ ^41^ ^]^ Consistent with these studies, we first revealed that excessive lactate concentration resulted in NPC dysfunction and IVDD progression through ScRNA‐seq analysis and validation experiments. Based on the results above, we concluded that under unfavorable stimuli such as inflammation or aging, abnormal internal pressure and extracellular matrix tissue permeability within the IVD would result in the abnormal activation of glycolysis in NPCs and obstruction of lactate transport out of the NP tissue. In the early stages, enhanced glycolysis can be considered as a response of NPCs to harmful factors to restore IVD homeostasis. Nevertheless, the persistent excessive accumulation of lactate adversely affects NPC function.

Importantly, we firstly revealed that lactate caused the dysfunction of NPCs via the activation of ferroptosis. Ferroptosis is a non‐apoptotic form of cell death characterized by excessive lipid peroxidation.^[^ ^42^ ^]^ Ferroptosis is a known major contributor to IVDD, and that inhibiting ferroptosis can prevent its development.^[^ ^43^ ^]^ Furthermore, ACSL4 was found to be significantly upregulated following lactate administration. ACSL4 is a critical initiator of ferroptosis with the provision of PUFA‐containing phospholipid substrates and can catalyze the esterification of AA into PEs, of which AA‐PE is the primary substrate for iron‐induced peroxidation in ferroptosis.^[^ ^42^ ^]^ Lipidomic analysis further indicated that silencing ACSL4 could decreased the production of PE (C18:0–C20:4) and PE (C18:0–C22:4) in the presence of lactate. Therefore, lactate can activate and increase the susceptibility of NPCs to ferroptosis by enhancing ACSL4–PU–phospholipids peroxidation axis and that inhibiting the expression of ACSL4 can be an alternative to treat IVDD under lactate environment.

PTMs regulate the function of proteins and shape the cellular phenotype to accommodate with the environment. Recently, a novel type of PTM mediated by lactate, lysine lactylation (Kla), was reported to add a lactyl group to the epsilon amino group of lysine.^[^ ^32^ ^]^ However, it remains unclear whether lactylation regulates ACSL4 expression and function. As previous studies reported, the promotive effects of lactate on gene expression depended mainly on histone lactylation.^[^ ^30^ ^]^ We firstly investigated whether lactate regulated the transcription of ACSL4 via histone, and found that the increase in H3K18 lactylation in lactate‐treated NPCS was the most significant. Subsequent genome‐wide CUT&TAG analysis indicated the elevated level of H3K18la at promoters of ACSL4 in lactate‐induced NPCs. Furthermore, we also examined the possible lactylation of ACSL4, and identified the lactylation of the K412 site of ACSL4. Combined with the results of in vitro experiments, we could conclude that the K412 site could be a potential regulatory site for ACSL4 in the presence of lactate. Collectively, lactate can activate ferroptosis in NPCs via two main pathways: increasing the gene expression of ACSL4 via H3K18la and promoting the protein lactylation of ACSL4.

Remarkably, we also observed that lactate decreased the expression of SIRT3 via miR‐708‐5p, indicating the involvement of lactate‐induced IVDD. In our previous study, we have proved that SIRT3 was involved in the progression of IVDD by regulating oxidative stress‐induced ferroptosis in NPCs.^[^ ^43^ ^]^In our previous study, we found that the expression of SIRT3 decreased with the progression of IVDD. In addition, mice with *SIRT3* knocking out showed increased susceptibility to ferroptosis activation, whereas mice with *SIRT3* overexpression showed delayed IVDD and ferroptosis inhibition induced by USP11 deficiency.^[^ ^43^ ^]^ Notably, our previous study aimed to investigate the effects of USP11‐SIRT3‐ferroptosis axis on IVDD, whereas the regulatory mechanism of SIRT3 in ferroptosis remains elusive. A recent study has reported that the SIRT3 was a critical eraser to remove the lactyl moiety from lysine.^[^ ^44^ ^]^ Through molecular docking and in vitro experiments, we demonstrated that SIRT3 binds well to ACSL4 via the formation of multiple hydrogen bonds, and silencing or overexpression of SIRT3 increased or decreased the lactylation of ACSL4, respectively. Our current study further revealed the intrinsic mechanism of SIRT3 in regulation ferroptosis from a perspective of ACSL4 lactylation.

Modulation of metabolic actions has been recommended for the treatment of various diseases such as OA, tumor response to chemotherapy, and immune regulation.^[^ ^45^, ^46^ ^]^ In OA, inhibition of glycolysis showed a protective effect against chondrocyte degeneration.^[^ ^47^ ^]^ Manoj et al.^[^ ^48^ ^]^ noted that LDHA inhibition by FX11 could reduce catabolic activity and oxidative stress, indicting the importance of metabolic–inflammatory axis in regulating OA. However, studies of metabolic modulation and IVDD are limited. To explore the therapeutic effects of inhibiting lactate production in IVDD, we chose the needle‐induced IVDD model via two kinds of mice experiments. There were several reasons for us to choose needle‐induced IVDD mice model. First, mice are widely used in biomedical research due to the availability of extensive genetic tools, such as mice with LDHA inhibition used in our research, which enabled the precise investigation of molecular mechanisms in IVDD. Second, although the needle‐induced IVDD model is more commonly used in rats, it also has been successfully established in mice.^[^ ^49^ ^]^ For example, Gao et al. demonstrate that needle puncture in mouse tail discs reliably induces degenerative changes consistent with human IVDD.^[^ ^50^ ^]^ Thirdly, mice are smaller, cost‐effective, and easier to handle, allowing for larger sample sizes and high‐resolution imaging. These practical benefits can enhance the feasibility of detailed histological and molecular analysis.^[^ ^49^ ^]^ Finally, the mouse model shared similar features to human IVDD, such as mechanical injury and inflammation, making it suitable for therapeutic research.^[^ ^51^, ^52^ ^]^ As shown in the results, either gene silencing or chemical inhibition markedly attenuated injury‐induced IVDD by suppressing lactylation and ferroptosis. These encouraging treatment outcomes highlight the reduction of lactate production is a promising therapeutic strategy for IVDD.

This study has several limitations. First, transcriptomics is good at showing the changes in expression of related genes and may be limited to using the results of transcriptomics to reflect metabolic remodeling. Therefore, metabolomic analysis of human IVD tissues with different Pfirrmann scores is required, and the results will more directly reveal the correlation between metabolic alterations and IVDD. Second, metabolic reprogramming can be a response of human NPCs to internal or external stimulation, and searching for critical upstream pathways or genes that regulate metabolism within cells is recommended. Third, NPC differentiation is a complex process and other energy metabolism‐ or aging‐related pathways participate in this process. This study focuses on glycolysis and IVDD. In future studies, we will investigate changes in other energy metabolism‐ and aging‐related pathways under IVDD. Fourth, the injection dose of AAV9 used into mouse IVD tissue varies in different studies.^[^ ^53^, ^54^ ^]^ Although the satisfactory therapeutic effects using AAV9 in this study have confirmed that injection volume (1 µL) was well‐tolerated and effective for delivering therapeutic agents, the optimum dose of AAV9 injection into the mouse IVD tissue remains further investigating to achieve optimal therapeutic outcomes and to minimize the potential toxic side effects of AAV9. Finally, 15% FBS for passage 0 NPCs can be a potential limitation of our study, as it may alter the phenotype or behavior of NPCs compared to their native state within the IVD tissue. We will further design our studies to explore alternative culture systems, such as low‐serum or serum‐free media supplemented with growth factors, to better mimic the physiological environment of IVD tissue.

## 4 Conclusion

Collectively, under unfavorable stress conditions, NPCs undergo metabolic remodeling, and glycolysis is abnormally activated. Excessive production and accumulation of lactate promote gene transcription and lactylation of ACSL4 to enhance ferroptosis, and lactate‐induced increased level of H3K18la and decreased expression of SIRT3 further results in the elevation of ACSL4 lactylation. Nevertheless, inhibiting glycolysis via gene silencing or chemical intervention reduces the production of lactate and ameliorates ferroptosis activation and NPC dysfunction during IVDD. The results of this study suggest the vital role of lactate‐ferroptosis axis in mediating IVDD, and targeting lactate production and/or lactate transmembrane transport could be a new therapeutic approach for patients with IVDD.

## 5 Experimental Section

### Single Cell Transcriptome Sequencing (scRNA‐seq) Data Acquisition and Analysis

The scRNA‐seq data pertaining to NP tissues from patients with IVDD were sourced from the NCBI Gene Expression Omnibus (GEO) database (GSE165722).^[^ ^21^ ^]^ The detailed information for the samples is shown in Table S1 (Supporting Information). scRNA‐seq was performed using the Rhapsody system (BD Biosciences). The Seurat package was used to process the scRNA‐seq data.^[^ ^55^ ^]^ Briefly, cells containing low‐quality data, specifically those with fewer than 200 or more than 6000 expressed genes, were excluded because barcodes showing abnormal gene counts could suggest the presence of dying cells, cells with compromised membranes, or doublets. In addition, cells were discarded in which over 40% of unique molecular identifiers were associated with mitochondrial genes, as an excessively high proportion of mitochondria often indicated leaky cytoplasmic membranes, allowing mRNA leakage. After filtering out the low‐quality cells, the top 2000 variable genes for principal component analysis (PCA) and the 30 most significant principal components for cluster analysis were selected. To mitigate batch effects, the “Runharmony” function from the Harmony package, which integrated scRNA‐seq data from multiple samples, was used. Subsequently, the clusters were visualized using a UMAP. Cells were categorized based on the expression patterns of classical markers (Figure S1, Supporting Information).

For the scRNA‐seq analysis, after NPCs were cultured with or without lactate (10 × 10^−3^ m) or CHC (a classical inhibitor for lactate transport) for 24 h, the cells were collected and subjected to scRNA‐seq on 10× Genomics Chromium System. Cell Ranger software was used for alignment and quantification of the raw data. The following analysis is consistent with the above descriptions. NPCs were divided into four groups according to their surface marker genes: MKI67^+^Serpine1^+^, MKI67^+^ATF4^+^, SLC38A1^+^, and COL1A1^+^ NPCs. All cell clusters were annotated using CellMarker (http://bio‐bigdata.hrbmu.edu.cn/CellMarker/).

### Pseudo‐Time Analysis of NP Clusters

A pseudo‐time analysis of the NPC timing sequence in each sample was conducted using the Monocle 2 package.^[^ ^56^ ^]^ For clustering, differential expression analysis was performed on genes expressed in more than ten cells. Pseudo‐time‐dependent gene expression across all NPCs was analyzed in an unsupervised manner. A heatmap was used to visualize the expression of all pseudo‐time‐dependent genes. According to the pseudo‐time, the cells were plotted along the trajectory and colored according to their cluster identities. GO and KEGG analyses of the gene modules that changed dynamically with pseudo‐time were conducted using the Cluster Profiler package.^[^ ^57^ ^]^

### Estimation of Signature Score for scRNA Cell Clusters

To assess the signature score in different cell subclusters, pathway genes were first obtained from the MsigDB dataset (https://www.gsea‐msigdb.org/gsea/msigdb/), which is a collection of annotated gene sets. The ferroptosis gene set was obtained from the FerrDb database (http://www.zhounan.org/ferrdb/current/) and included 370 ferroptosis driver genes and 349 ferroptosis inhibitor genes. Genes that were not expressed in the scRNA‐seq data were excluded. The “AddMouduleScore” function in Seurat was then used to assess the scores for the selected pathways. The “geom_smooth” function in ggplot2 package was used to show the dynamic changes in pathway score with pseudo‐time.

### Collection of Clinical Samples

NP samples were harvested from 20 patients (9 female and 11 male patients, mean age: 53.6 ± 17.3) who underwent lumbar discectomy for lumbar degenerative diseases, tethered cord syndrome, lumbar spondylolysis, or fracture‐related diseases. Written informed consent was obtained from all the patients. Based on preoperative magnetic resonance imaging (MRI) and the Pfirrmann score, the NP samples were divided into four groups: Grades II (*n* = 5), III (*n* = 5), IV (*n* = 5), and V (*n* = 5). The clinical data for the patients are shown in Table S2 (Supporting Information).

### Isolation and Culture of Human NPCs

Briefly, after collecting the human IVD tissue (Grade II), fresh gel‐like NP tissue was selected and digested sequentially with trypsin (0.25% EDTA+) (cat. no. 25200072; Gibco, Thermo Fisher Scientific) for 30 min, and collagenase type II (0.2%) (Sangon Biotech, cat. no. A004174‐0100) for another 60 min in a 37 °C water bath. Subsequently, the isolated NPCs were resuspended in DF‐12 culture medium (cat. no. 11330057; Gibco, Thermo Fisher Scientific) supplemented with 15% FBS (cat. no. 10099‐141C; Gibco) and 1% penicillin–streptomycin (cat. no. 15140163; Gibco, Thermo Fisher Scientific). The passage 0 NPCs were then cultured in an incubator at 37 °C. When the passage 0 NPC density reached ≈90%, the samples were digested and prepared for subsequent experiments. Notably, the following passaging NPCs would be cultured with 10% FBS.

### RNA Reverse Transcription and Quantitative Real‐Time Polymerase Chain Reaction (RT‐qPCR)

Initially, the total mRNA was collected from NPCs and then the HiScript III RT SuperMix for qPCR Kit (cat. no. R323‐01; Vazyme) was utilized to reverse transcribe mRNA into cDNA. Next, the SYBR qPCR Master Mix (cat. no. Q711‐02; Vazyme) was used to visualize the relative expression levels of the obtained cDNA. β‐Actin was used as the reference gene. Relative gene expression was quantified using the formula: 2^−△△Ct^. Primers used in this study are presented in Table S3 (Supporting Information).

### Cell Viability Assay

Cell viability was assessed using the CCK‐8 reagent (C0005, Topscience Biotechnology Co., Ltd., Shanghai, China), following the manufacturer's instructions. Briefly, primary human NPCs (4000 cells per well) were cultured in a 96‐well plate for 24 h. The NPCs were then co‐cultured with lactate (0.01, 0.1, 1, 10, and 20 × 10^−3^ m) or 2‐DG (0, 0.1, 0.5, 1, 10, and 100 × 10^−6^ m) for another 24 h. Next, the culture medium was removed, and NPCs were washed two times using PBS. Then, 110 µL of working solution (100 µL medium and 10 µL CCK8 solution) was added onto each well and further co‐incubated for 2 h at 37 °C. The absorbance was measured using an enzyme labeling device at 450 nm to detect cell viability.

### Histology Analysis

Histology analysis in this present study included human and mouse IVD tissues. Human NP tissue was acquired intraoperatively from the patients, and the mouse IVD tissue was collected at the terminal of the animal experiment. For histological analysis, including H&E staining and SOFG staining, the IVD tissues were firstly preserved in 4% paraformaldehyde for 3–7 d, and then they underwent decalcification process. In generally, human IVD tissue required a four‐week decalcification process, and the decalcification process of the mouse IVD required two weeks. Subsequently, the IVD tissues were subjected to paraffin‐embedding, and sagittal sections of 3–4 µm thickness were cut and stained with H&E and SOFG. The details of H&E and SOFG staining were described previously. The degree of human or mouse IVDD was evaluated using the two newly reported grading scales, respectively.^[^ ^22^, ^58^ ^]^ For human NP tissue, the new grading system^[^ ^22^ ^]^ includes the cellularity (score 0–3), lesions (tears, clefts, and voids) (score 0–3), and ECM structure (score 0–3) (Table S4, Supporting Information). For mouse IVD tissue, the new grading system^[^ ^58^ ^]^ provided a more detailed description regarding the subfeatures of IVD tissue, which was established based on naturally occurring mouse IVD pathologies, the previous scoring systems and feedback received from the spine community (Table S5, Supporting Information). The score system for mouse IVDs was classified using a point‐based ordinal scale of equal intervals (0, 1, 2, and 3) to separately grade NP, AF, EP, and the interphase regions. Adding the scores for features within a specific IVD region will inform about the pathology of that region where highest score will be 9 for NP, 12 for AF, 6 for EP, and 8 for the interface. The categories are linearly ordered with a score of 0 representing a normal structure, an increase in number scores increased histopathology, with the highest score indicating severe degeneration.

### Transcriptome Sequencing (mRNA‐seq) and Bioinformatics Analysis

Human NPCs were seeded onto six‐well (10^5^ cells/well) for 24 h to allow adhesion to the plate. Then, NPCs were treated with either IL‐1β (10 ng mL^−1^) or lactate (10 × 10^−3^ m) for another 24 h. Next, the total mRNA of NPCs was collected using TRIZOL solution and quickly frozen at −80 °C. The mRNA‐seq analysis of human NPCs was conducted in collaboration with Genekinder Medicaltech (China). Subsequently, the R package Deseq2 was used to identify and analyze DEGs.^[^ ^59^, ^60^ ^]^ Further analysis was performed on mRNAs that exhibited differential expression with a log2(FC) value greater than 1 and an FDR value less than 0.05. Visual representations of DEGs between the two samples are presented through volcano and heatmap plots generated using the ggplot2 and heatmap packages, respectively. Intersecting genes were visualized using a Venn diagram to represent shared ferroptosis genes across the bulk‐RNA‐seq and scRNA‐seq datasets.

### Lactate Measurement

After preparation, the lactate concentration was measured in human NPCs or IVD tissue using a lactate assay kit (BC2235; Solaribo) according to the manufacturer's instructions. Cell samples were incubated at room temperature for 30 min, and enzyme‐labeling devices were used to measure the absorbance at 570 nm.

### GSH/NADPH detection assay

NPCs were distributed in six‐well culture plates at a density of 10 × 10^6^ cells/well. After treatment, the cells were lysed on ice and the collected supernatant was used to determine the levels of GSH and NADPH using commercial kits according to the manufacturer's instructions. The GSH and NADPH kits were purchased from Beyotime Co., Ltd. (S0053 and S0058).

### siRNA for LDHA, ACSL4, or SIRT3 Transfection Assay

For silencing human ACSL4, SIRT3, and LDHA, we designed three small interfering RNAs (siRNAs) sequences for each gene with the help of GenePharma (Shanghai, China). Briefly, human or mouse NPCs were transplanted to 12‐well plates. After the cell density reached to 60%–70%, Lipofectamine 2000 Reagent (Invitrogen) was used to transfect the siRNA for ACSL4, SIRT3, or LDHA to NPCs following the manufacturer's protocol. The synthetic siRNA sequences for human *ACSL4*, human *SIRT3*, and mouse *Ldha* were presented in Table S6 (Supporting Information). After transfection for 24 h, the mRNA was collected and RT‐qPCR was used to verify the effect of gene silencing, and the siRNA that showed the most obvious ability to suppress the mRNA level of ACSL4, SIRT3, or LDHA was chose for the following experiment. Finally, *ACSL4*‐si‐3, *SIRT3*‐si‐1, and *Ldha*‐si‐2 were chosen as the best gene silencing siRNAs (Figure 7G and Figure S13, Supporting Information). The best sequence for human *ACSL4* was as following: GCAAAGAAGCAGUAGUUCATT (sense), UGAACUACUGCUUCUUUGCTT (anti‐sense), for mouse *Ldha* was as following: AGCAAAGACUACUGUGUAA (sense), UUACACAGUAGUCUUUGCU (anti‐sense), and for human *SIRT3* was as following: GGUGGAAGAAGGUCCAUAUTT (sense), AUAUGGACCUUCUUCCACCTT (anti‐sense).

### Plasmid Transfection

A previous study established the SIRT3 overexpression plasmid.^[^ ^43^ ^]^ Briefly, then the coding sequence (CDS) of Sirt3 (GenBank ID: AF083108.2) was obtained from National Center for Biotechnology Information (https://www.ncbi.nlm.nih.gov/). The full‐length sequence of SIRT3 was synthesized and subcloned into the Flag vector by GenePharma Biotechnology Co., Ltd. (Shanghai, China). Then, human NPCs were transfected with the plasmid using Lipo8000 reagent (C0533, Beyotime) according to the manufacturer's instructions. Briefly, the day before transfection, the cells were seeded in six‐well plates (about 5 × 10^4^ cells per well). The culture medium was changed when the cells reached 80% confluence. The mixture was added with the amount of 130 µL (125 µL of Opti‐MEM°R medium + 2.5 µg of DNA + 4 µL of Lipo8000 reagent) into each well, and incubated for 24 or 48 h. Finally, the mRNA or protein was collected for the following experiments.

### IP‐Mass Spectrometry

LC‐MS/MS analysis was supported by Jingjie PTM BioLabs (Hangzhou, China). Briefly, the human NPCs were incubated with co‐cultured with lactate (10 × 10^−3^ m) for 24 h. Then, the anti‐ACSL4 antibody (Clone#26A48, Boster) was used for IP assay. Immunoprecipitated proteins were subjected to electrophoresis by ExpressPlus PAGE Gel electrophoresis and brilliant Coomassie blue staining (P0003S, Beyotime). Enriched proteins were further analyzed for lactylation identification by tandem mass spectrometry (MS/MS) in Q Exactive^TM^ Plus (Thermo) coupled online to the UPLC. The resulting MS/MS data were processed using Proteome Discoverer 1.3. Trypsin/P (or other enzymes if any) was specified as cleavage enzyme allowing up to two missing cleavages. Mass error was set to 10 ppm for precursor ions and 0.02 Da for fragment ions. Carbamidomethyl on Cys were specified as fixed modification and oxidation on Met was specified as variable modification. Peptide confidence was set at high, and peptide ion score was set >20. According to the results of LC‐MS/MS analysis, only one significant lactylation site (K412R) was detected on ACSL4 (Figure 6A). Therefore, this study focused on the K412 site lactylation of ACSL4 in the following experiments.

### Untargeted Lipidomics Analysis

NPCs were seeded in six‐well culture plates at a density of 10 × 10^6^ cells per well and transfected with si‐NC or si‐ACSL4 using lip3000 for 4 h, followed by the administration of lactate (10 × 10^−3^ m) for another 20 h. Then, the cells were collected and quickly frozen at −80 °C. Lipids were detected using the Cosmos Wisdom Laboratory (Cosmos Wisdom, Co., Ltd., Hangzhou). Briefly, lipids were extracted from NPCs. Chromatographic separation of the target compounds was performed using a high‐performance liquid chromatography (HPLC) instrument with a Thermo Accucore C18 HPLC column (2.1 mm × 100 mm, 2.6 µm particle size). MS and MS/MS analyses of lipids were performed using a Q‐Exactive hybrid quadrupole‐Orbitrap mass spectrometer (Thermo Fisher Scientific). The relative parameters were IS 5200/‐4500, TEM 500, CUR 35, GS1 55, and GS2 55. Annotations, functional definitions, and classifications of all detected lipids were conducted based on the LIPID MAPS Lipidomics Gateway (https://www.lipidmaps.org/) and LION (Lipid Ontology) databases (http://lipidontology.com/). Visualization was performed using the ggplot2 package (version 3.4.4). Variables that simultaneously met the criteria of *p*‐value < 0.05, and variable importance in projection (VIP) > 1.0, were considered differential lipids.

### Molecular Docking

The structures of ACSL4 (ID: O60488) and SIRT3 (ID: Q9NTG7) were retrieved from UniProt (Universal Protein) and then subjected to the HDOCK server to simulate molecular docking of the ligand with proteins. For the two individual protein structures, HDOCK attempted to sample all possible binding modes between the two proteins. Subsequently, a scoring function was used to rank both the sampling and post‐sampling binding modes. Owing to the lack of information on the binding site, ab initio global docking had to be performed to sample the hypothetical binding modes across six degrees of freedom (three rotations and three translations). This process involved two indicators: the docking score (a more negative docking score indicating a more likely binding model) and confidence score (a value above 0.7 suggests a high likelihood of binding between the two molecules). Based on the Vina docking score, the best pose was selected and visually analyzed using PyMoL 1.7.6 (www.pymol.org).

### Dual Luciferase Report Assay

The binding sequences between miR‐708‐5p and SIRT3 were predicted using Target Scan software. The SIRT3 3′‐untranslated region (3′‐UTR) containing wild‐type (WT) or mutant (MUT) binding sites for miR‐708‐5p (BioTNT, Co., Ltd.) were inserted into a pmirGLO vector (Promega). Subsequently, the human SIRT3 3′‐UTR fragments and miR‐708‐5p mimics were co‐transfected into human NPCs using Lipofectamine 3000 (Invitrogen). A Dual Luciferase Assay Kit (Promega) was used to measure luciferase activities at 48 h after transfection. Renilla luciferase activity was used for normalization.

### Establishment of Needle‐Induced Mouse IVDD Model and Therapeutical Intervention

This method was described in detail in a previous study.^[^ ^61^ ^]^ For the AAV9 transfection assay, male mice (C57BL/6) with the age of 6–8 weeks were randomly divided into four groups (*n* = 5/group): sham surgery, IVDD, IVDD+ AAV9‐NC, and IVDD+ AAV9‐si‐*Ldha*. After adaptation to the environment for 7 d, the mice underwent sham or needle puncture‐mediated IVDD surgery. For treatment group, the mice received the intradiscal injection of either AAV9‐NC or AAV9‐si‐*Ldha* (1 µL per mouse). For the chemical glycolysis inhibition assay using 2‐DG, a glucose analog, mice were divided into three groups (*n* = 5/group): sham, IVDD, and IVDD+2‐DG (CAS154‐17‐6; TOPSCIENCE) (intraperitoneal injection, 0.5 g kg^−1^, twice a week for the first two weeks).^[^ ^62^ ^]^ Eight weeks after surgery, the IVD tissues were collected and evaluated radiologically and histologically.

To establish the IVDD mouse model, mice were anesthetized with 2.5% inhalational isoflurane and positioned prone on a warming pad maintained at 37 °C. The tails were sterilized and the intervertebral space was located by palpation. Based on the anatomical characteristics of mouse IVD, the bony structures at the vertebral ends of the IVD in the caudal vertebrae exhibit a slightly widening trend. When gently sliding along the axial direction of the mouse tail using the thumbnail for palpation, a noticeable pause can be felt at the location of the intervertebral space, indicating the position of the disc. When the thumbnail slides to the position of the intervertebral space, a noticeable skin depression can be observed at this location. A needle of the sterile syringe (2 mL, the needle size of a 2 mL sterile syringe is about 25G) was used to make a perpendicular puncture into the tail disc until the center of the NP tissue was reached. The needle was then rotated 180° and held in place for 30 s. Notably, since the diameter of the mouse tail vertebra was ≈3 mm, the needle used would be affixed using a clamp as a depth stop so that a 1.5 mm tip stuck out to ensure the needle tip reaches the center of the nucleus pulposus tissue. In addition, during the procedure of puncture, the needle did not penetrate the whole disc tissue but only reached the center of the nucleus pulposus. No aspiration was performed during the process. However, since the needle is hollow, a small amount of nucleus pulposus tissue may be carried out by the needle. The mice were housed in an animal room with a constant temperature of 20–22 °C and humidity of 50%–65%, where they had free access to food and water. The light–dark cycle was maintained for 12 h. Eight weeks postoperatively, the mice were euthanized and their IVD tissues were collected for subsequent histochemical experiments.

### Adeno‐Associated Virus 9 (AAV9) infection

To inhibit the in vivo expression of mouse *Ldha* in vivo, the critical enzyme for the production of lactate, siRNAs were designed for mouse *Ldha*. After the best siRNA sequence for si‐ *Ldha* had been confirmed in the former experiments (3′‐AGCAAAGACUACUGUGUAA‐5′; 3′‐UUACACAGUAGUCUUUGCU‐5′), AAV9 that carried si‐*Ldha* was designed to silence *Ldha* (GenePharma, Shanghai, China). AAV9‐NC was selected as control vector (GenePharma, Shanghai, China). Briefly, C57 mice (6–8 weeks) were firstly anesthetized with 2% isoflurane and oxygen. Then, the Co6/7 (caudal tail disc 6/7) disc was identified by palpation and this position was cleaned with alcohol. Subsequently, a 5 µL micro‐injection syringe (MICROLITER Series; Hamilton Bonaduz, Switzerland) was used to inject AAV9‐si‐*Ldha* (1 × 10^12^ GC mL^−1^, 1 µL) into the IVD tissue after the model had been constructed. After leaving the needle in place for 5 s, the needle was carefully removed without excessive mechanical disruption of the NP tissue.

### Micro‐Computed Tomography

At eight weeks after surgery, micro‐CT was performed on all mice before sacrifice. After anesthetization, the mice were placed in the prone position with their spine straight. The micro‐CT data were reconstructed and converted into three‐dimensional images using NRecon, Dataviewer, and CTvox software. The disc height index (DHI%) was calculated by normalizing the disc height to the height of the adjacent vertebral body.

### Immunohistochemistry (IHC) and IF staining

For IHC staining, the deparaffinized and hydrated histological slides of human NP tissue were blocked by 5% bovine serum albumin (BSA) for 30 min at room temperature and incubated overnight (4 °C) with primary antibodies against LDHA (GB11342; Servicebio), HK2 (GB111063; Servicebio), and G6PD (GB111797; Servicebio). Next, the slides were washed with TBST (G0004; Servicebio), followed by the incubation with secondary antibodies, HRP‐conjugated goat anti‐rabbit IgG (GB23303; ServiceBio). For IF staining of IVD tissue, the deparaffinized and hydrated histological slides of human NP tissue were blocked with 5% BSA for 30 min at room temperature and incubated overnight (4 °C) with primary antibodies against Pan‐Kla (PTM‐1401; PTM BIO, China; 1:100), ACSL4 (DF12141; Affinity), 4‐HNE (bs‐6313R; Bioss), and ACAN (DF7561, Affinity). For the IF staining of human primary NPCs, after treatment, the cells were fixed using 4% paraformaldehyde for 15 min, permeabilized using 0.15% Triton X‐100 for another 5 min, washed three times with PBS, and then incubated with LDHA (GB11342; Servicebio), HK2 (GB111063; Servicebio), ACAN (DF7561; Affinity), MMP3 (AF0217; Affinity), 4‐HNE (bs‐6313R; Bioss), SIRT3 (AF5135; Affinity), and ACSL4 (DF12141; Affinity). Next, the slides or NPCs were washed with TBST (G0004; Servicebio), followed by incubation with the secondary antibodies, FITC/Cy3 IgG (GB21401 and GB22301; Servicebio). Finally, a microscopic examination was performed. Semi‐quantitative intensity was analyzed using the ImageJ software.

### Statistical Analysis

In this study, independent experiments or repeated measurements were conducted with sample sizes of three, four, or five. Data are presented as mean ± standard deviation (SD). GraphPad Prism 9 (GraphPad Software Inc., La Jolla, CA) was used for statistical analysis. Normality hypothesis and the homogeneity of variance of data were determined by Shapiro's test and Levene's test before comparisons. To determine significant differences between groups, two‐tailed unpaired Student's *t*‐tests and one‐way analysis of variance (ANOVA) were used followed by Tukey's post hoc test. A *p*‐value less than 0.05 would be considered statistically significant (*P < 0.05, **P < 0.01, ***P < 0.001, and ****P < 0.0001).

### Ethics Statement

The use of human tissues was approved by the Institutional Human Ethics Review Board of the Naval Medical Center, with approval number 2023030310‐2 (March 10, 2023). In addition, authorization to conduct experiments involving animal subjects was granted by the Naval Medical Center, with approval number NMC‐2023031‐2 (March 10, 2023).

## Conflict of Interest

The authors declare no conflict of interest.

## Supporting information



Supporting Information

Supporting Information

Supporting Information

## Data Availability

Our detailed single‐cell RNA‐seq data are deposited at Mendeley Data (https://data.mendeley.com/datasets/bbmh79cwrt/1). Other data, including IP‐mass spectrometry, needed to evaluate the conclusions in the paper are present in the paper and/or the Supporting Information. Additional data related to this paper may be requested from the authors.
