# Supplementary material for: Glycolysis‐Derived Lactate Induces ACSL4 Expression and Lactylation to Activate Ferroptosis during Intervertebral Disc Degeneration
Source: Adv Sci (Weinh). 2025 Apr 2;12(21):2416149. doi: 10.1002/advs.202416149 (PMC12140309; doi:10.1002/advs.202416149)
Supplement: Supplementary file 1 — Supporting Information [file ADVS-12-2416149-s003.docx]

Supporting information

**Glycolysis-derived lactate induces ACSL4 expression and lactylation to activate ferroptosis during intervertebral disc degeneration**

Kaiqiang Sun^ψ^, Yangyang Shi^ψ^, Chen Yan^ψ^, Shunmin Wang^ψ^, Linhui Han, Fudong Li, Ximing Xu, Yuan Wang, Jingchuan Sun*, Zijian Kang*, Jiangang Shi*

ψ These authors have contributed equally to this work.

*Corresponding Author: Jiangang Shi, Zijian Kang, and Jingchuan Sun

**This file includes:**

Experimental sections

Figs. S1 to S13

Tables S1 and S7

**Experimental sections**

*Protein extraction and western blot analysis* Human NPCs were lysed after preparation using Super-RIPA lysis buffer with protease (P1005; Beyotime, Shanghai, China) and a phosphatase inhibitor cocktail (C0002; TOPSCIENCE). After quantification, total NPC protein was loaded onto a sodium dodecyl sulfate-polyacrylamide gel (10%, PG112; Shanghai Epizyme Biomedical Technology Co., Ltd, China) and transferred onto PVDF membranes (HVLP04700; Millipore, Sigma-Aldrich). After blocking the membranes with 5% dry skim milk, they were incubated with primary antibodies, followed by incubation with secondary antibodies. Antibodies included HK2 (GB111063; Servicebio, Wuhan, China), G6PD (GB111797; Servicebio), LDHA (GB11342; Servicebio), ACSL4/FACL4 Antibody (DF12141; Affinity), ACAN (GB11373; Servicebio, or ab186414; Abcam), Col2A1(ab34712; Abcam), MMP3 (340612; Zenbio), ADAMTS4 (DF6986; Affinity), SIRT1 (BF0189, Affinity), SIRT2 (AF5256, Affinity), SIRT3 (ab246522, Abcam), HDAC1 (AF6433), HDAC2 (AF6470, Afffinity), HDAC3 (AF6016, Affinity) and β-Actin (200068-8F10; Zenbio). Notably, during the design of this research, we have carefully learned previously published studies regrading glycolysis and lactylation, and found that up to date, totally 461 articles were identified, including 193 articles using beta-actin as the endogenous control, 36 articles using Tubulin as endogenous control, and 129 articles using GAPDH as endogenous control. The results indicated that β-actin was the most used in lactylation-related researches. Therefore, β-actin was used as the housekeeping gene or protein in our study.

(GSEA) was performed using the curated gene set of the two groups to identify differential GO/KEGG pathways. GSEA enrichment analysis were conducted using MSigDB gene sets (<https://www.gsea-msigdb.org/gsea/msigdb>).

*Analysis of mitochondrial membrane potential* According to the instructions, JC-1 staining was performed using a JC-1 staining kit (Beyotime Biotechnology, Inc., Shanghai, China).

*Evaluation of extracellular metabolic flux by Seahorse Assay* Human NPCs (1 × 10^4^/well) were cultured onto the XF96 cell culture microplate and treated with IL-1β (10 ng/mL) for 24 h. For extracellular acidification rate (ECAR), washed cells were kept in the pre-warmed XF assay media supplemented with 1 mM sodium pyruvate and 2 mM L-glutamine. At indicated time-points, XF assay media with 10 mM glucose, 2 μM oligomycin, and 50 mM 2-DG in were added. For mitochondrial respiration measurement, NPCs were incubated in glucose-containing culture medium for 1 h, and OCR was measured every five minutes before and after sequential addition of 1 μM oligomycin, 1 μM FCCP, 1 μM Rotenone, and 5 μM Antimycin A. The acquired data were analyzed using XFe96 software (Agilent Technologies).

*Transmission electron microscopy (TEM) assay* After treatment, NPCs were collected and stored in an electron microscope fixative (G1102; Servicebio) for approximately 2 h at room temperature. The fixed NPC samples were then subjected to post-fixing, dehydration, drying, and conductive metal coating. Next, an elemental analysis of the area of interest with ferroptotic mitochondria was performed and counted under a scanning electron microscope (SU8100; HITACHI).

*Lipid peroxidation (LPO) assay* Lipid peroxidation is a classic indicator of ferroptosis. NPCs were seeded into 6-well plates and stimulated with lactate (10 mM) with or without ACSL4 silencing. After 24 h, NPCs were incubated with 2 μL of Liperfluo probe (10 μm; MX5211-1MG) for 30 min at 37 °C. Finally, changes in LPO were observed by imaging under a fluorescence microscope (Olympus, Japan). Changes in lipid peroxidation were standardized by measuring the green-to-red fluorescence ratio.

*Intracellular Fe^2+^ level measurement* NPCs were seeded in a 12-well plate, and the cells were treated with lactate with or without Fer-1(T6500; TOPSCIENCE) for 24 h. Intracellular Fe^2+^ levels were evaluated according to the instructions of the FeRhoNox-1 fluorescent probe kit (MX4558; MaoKang Biotech, Shanghai, China). Briefly, after treatment, NPCs cells were stained in 1 μM FerroOrange (Dojindo, Kumamoto, Japan) in DF-12 culture medium for another 30 min at 37°C and then captured immediately via a fluorescence microscope (Leica DMI8, Weztlar, German).

**Supplementary figures and legends**


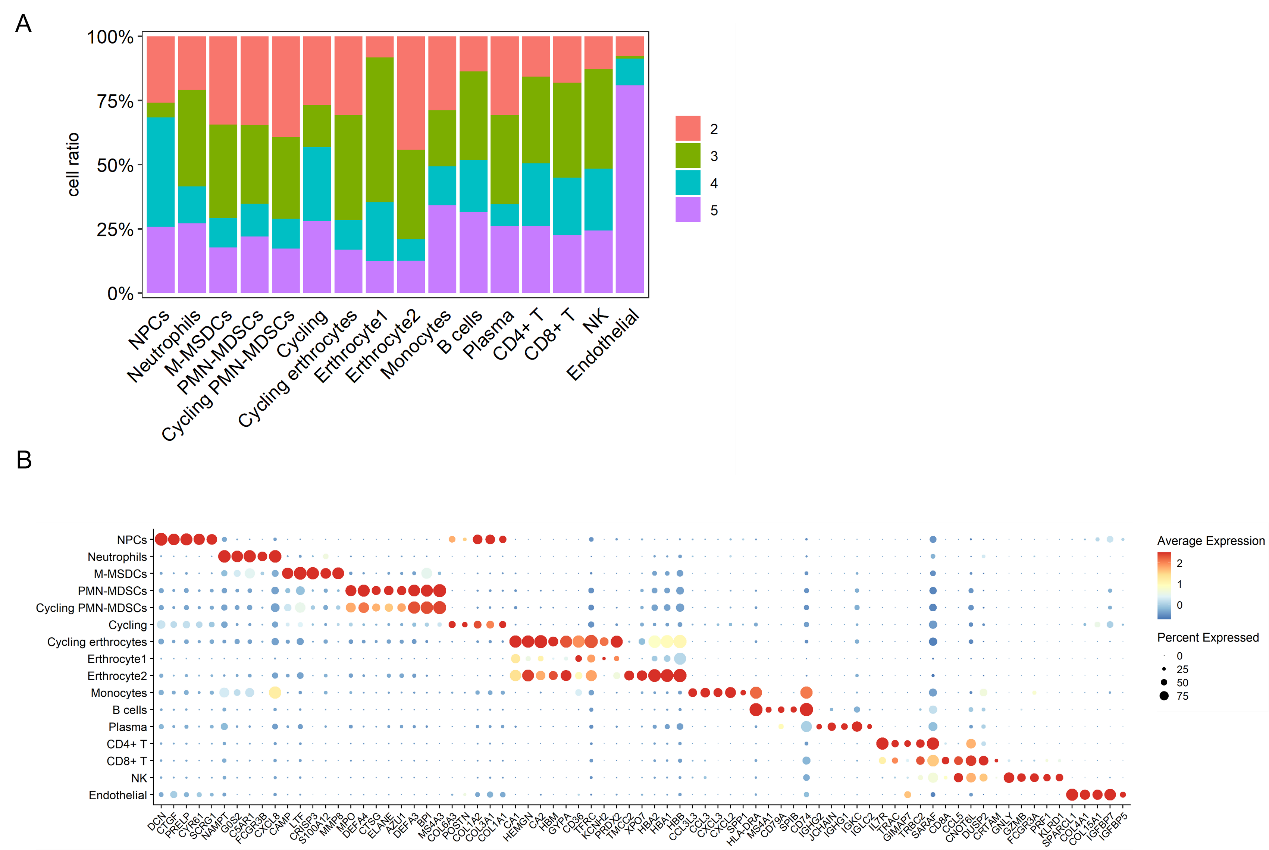


**Figure S1** A: Relative proportion of each cell clusters within human IVD tissue with different Pfirrmann score. B: Representative molecular signatures for each cell cluster. The area of the bubbles indicates the proportion of cells expressing the gene, and the color intensity reflects the expression intensity. IVD: Intervertebral disc.


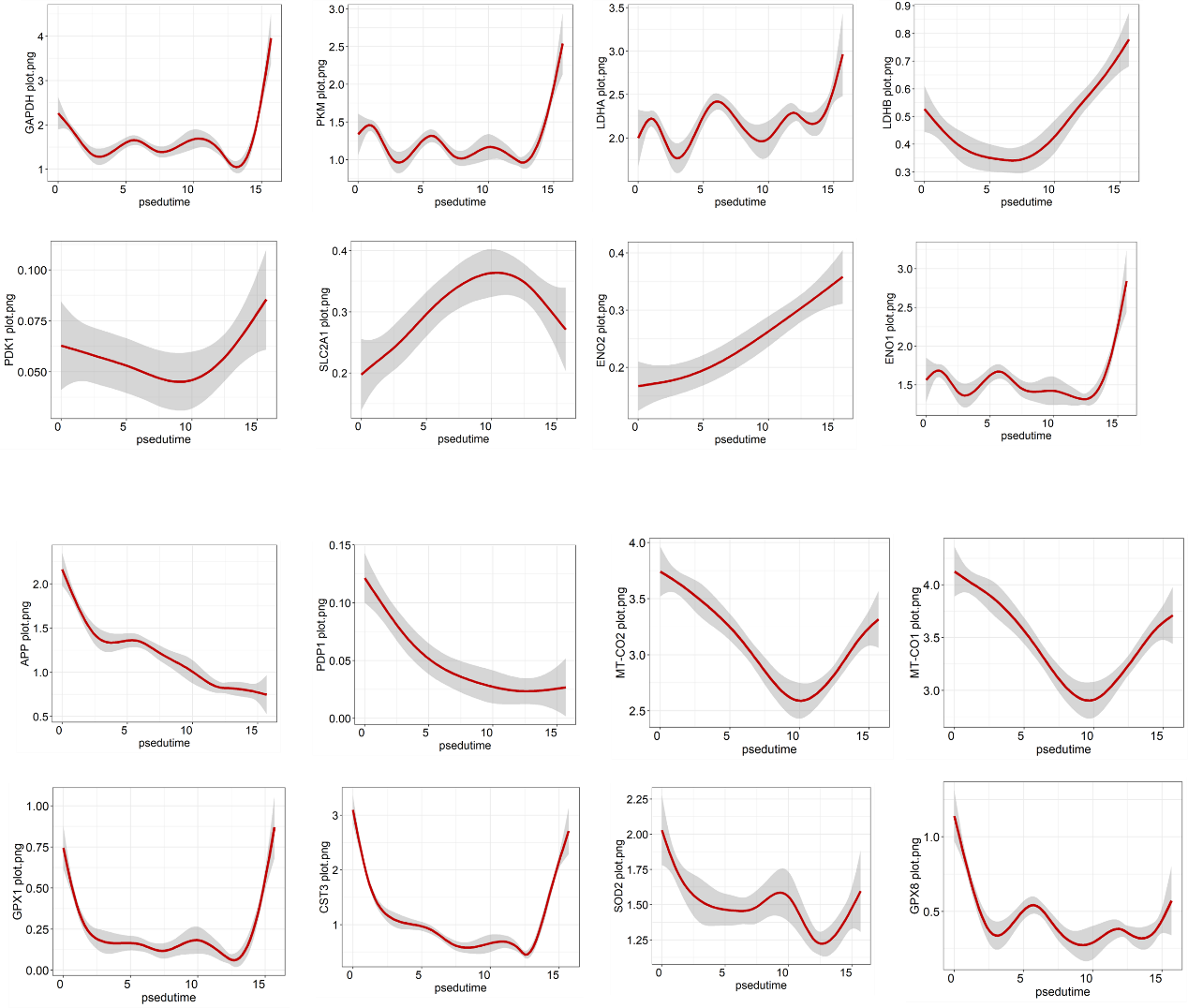
 **Figure S2 Signature score of the representative genes related to metabolism for energy metabolism of human NP cells. NP: Nucleus pulposus.**


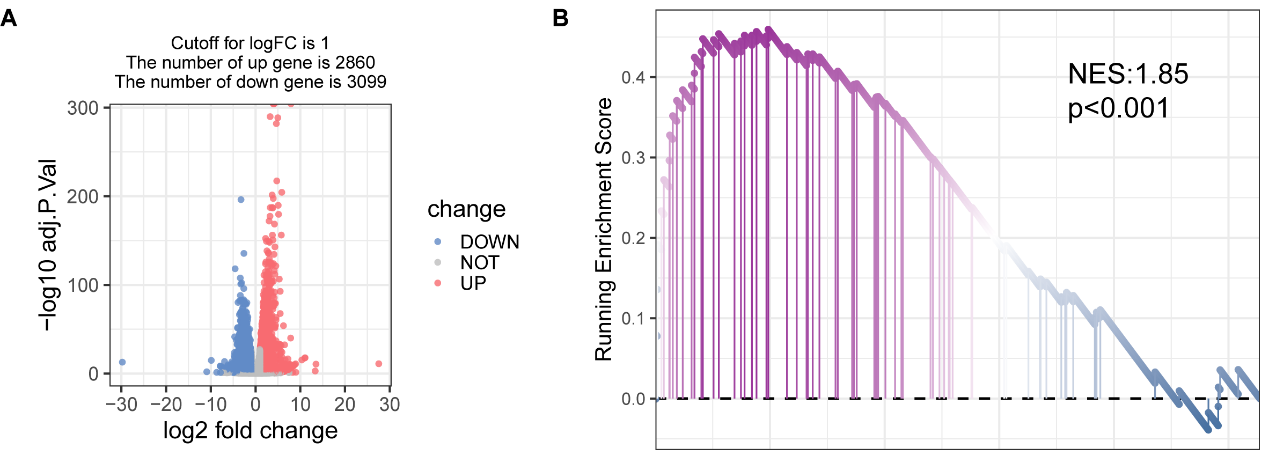


**Figure S3** A: The results of Volcano plot indicating all the differentially expressed (upregulated and downregulated) genes in lactate-treated rat NPCs (GSE219145, n=3). B: The results of GESA indicating activated ferroptosis signaling pathways in lactate-treated rat NPCs. GSEA: Gene Set Enrichment Analysis Gene set enrichment analysis; NPCs: Nucleus pulposus cells.


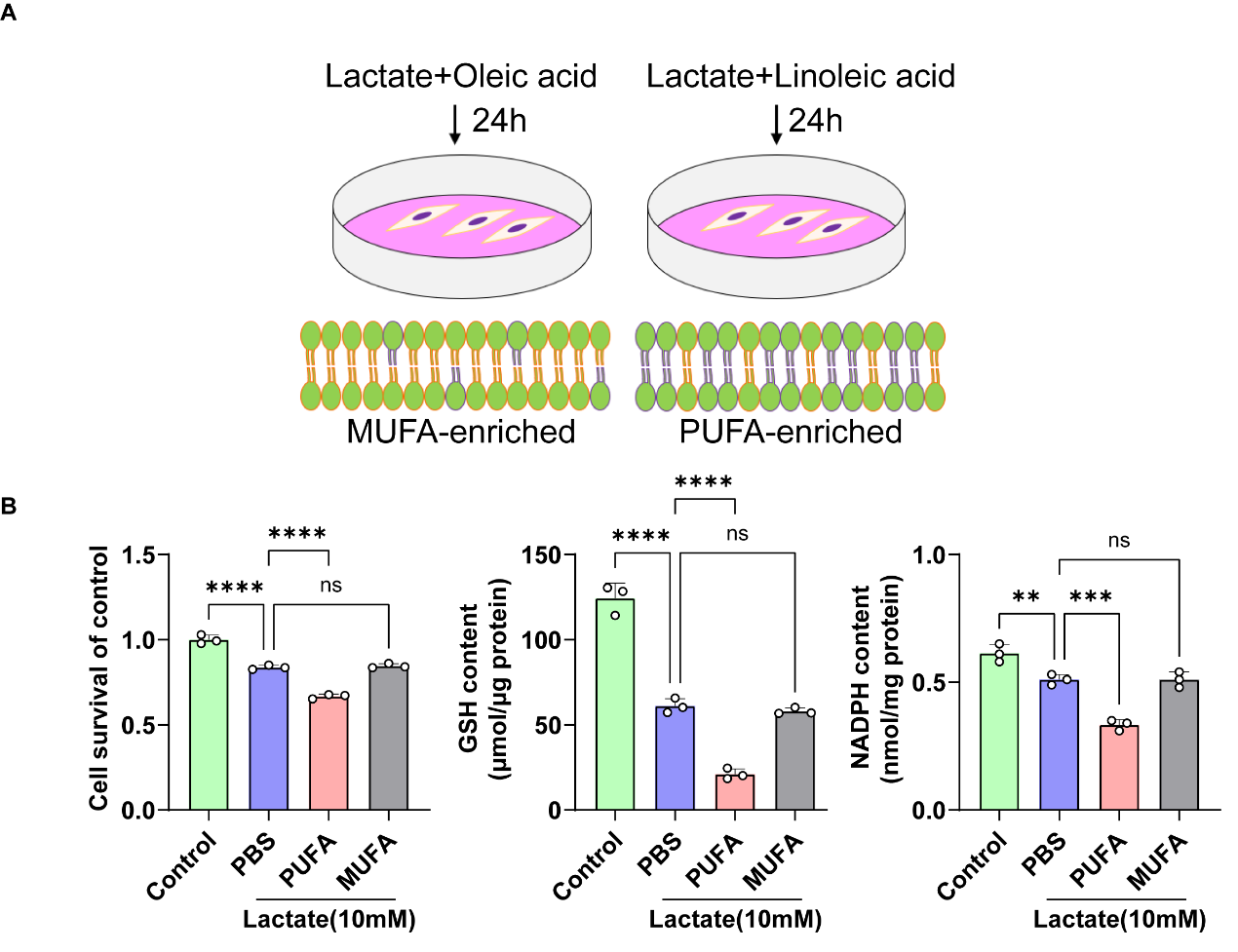


**Figure S4** A: Experimental schema for establishing MUFA or PUFA-enriched NPCs. NPCs were treated with oleic acid (OA, 80 μM) or linoleic acid (LA, 80 μM) cocultured with lactate for 24 h. MUFA, monounsaturated fatty acids; PUFA, polyunsaturated fatty acids. B: Cell survival and the content of GSH and NADPH in lactate-treated human NPCs in MUFA or PUFA-enriched environment for 24 hours. All data are shown as the mean ± SD. *P < 0.05, **P < 0.01, ***P < 0.001, ****P < 0.0001. MUFA: Monounsaturated fatty acids; PUFA: Polyunsaturated fatty acids. GSH: Glutathione; NADPH: Nicotinamide adenine dinucleotide phosphate; NPCs: Nucleus pulposus cells.


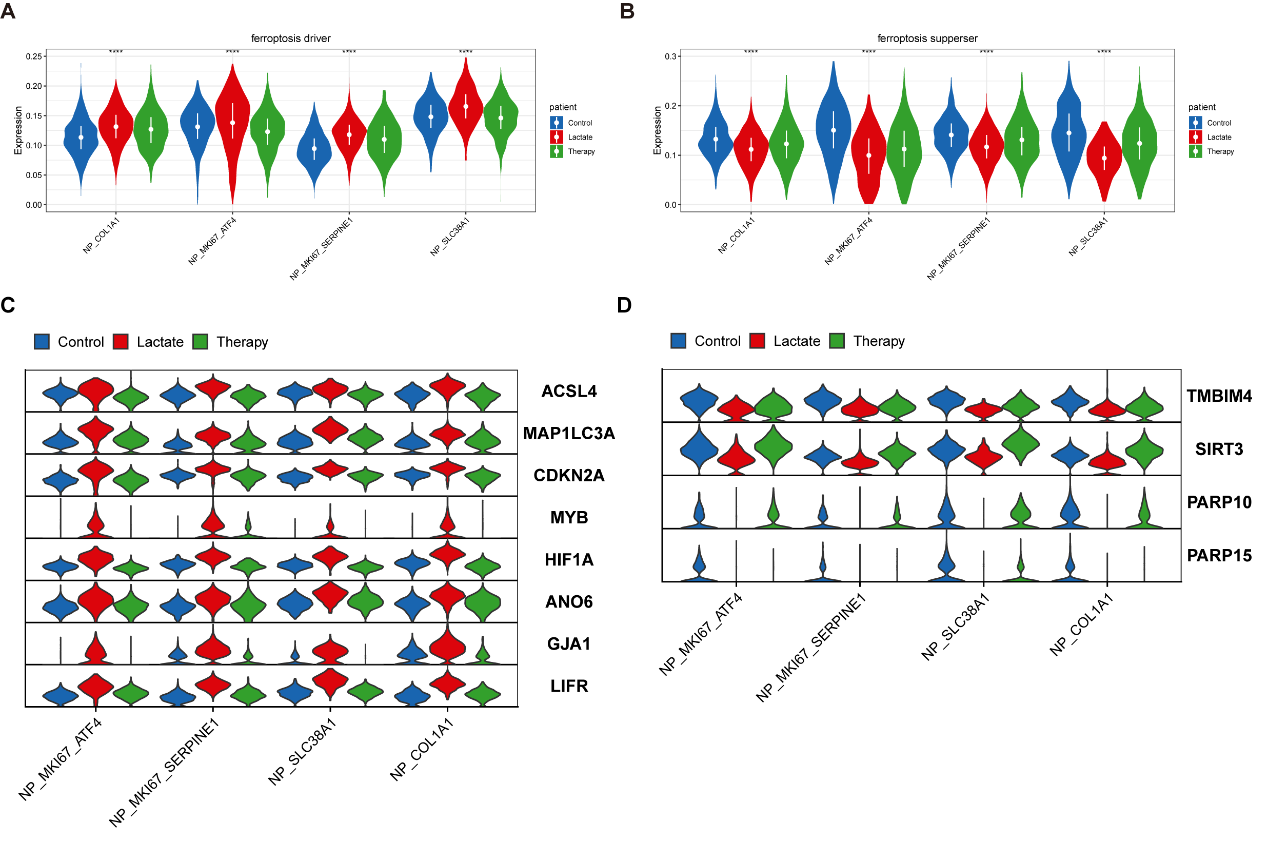


**Figure S5** A: The expression strength of ferroptosis driver-related genes between control group, lactate group, and lactate+CHC (Therapy) group. J: The expression strength of ferroptosis suppresser-related genes between control group, lactate group, and lactate+CHC (Therapy) group. C and D: Differentially expressed ferroptosis-related genes via integrated analysis of bulk RNA-seq and scRNA-seq between control group, lactate group, and lactate+CHC (Therapy) group. All data are shown as the mean ± SD. *P < 0.05, **P < 0.01, ***P < 0.001, ****P < 0.0001.


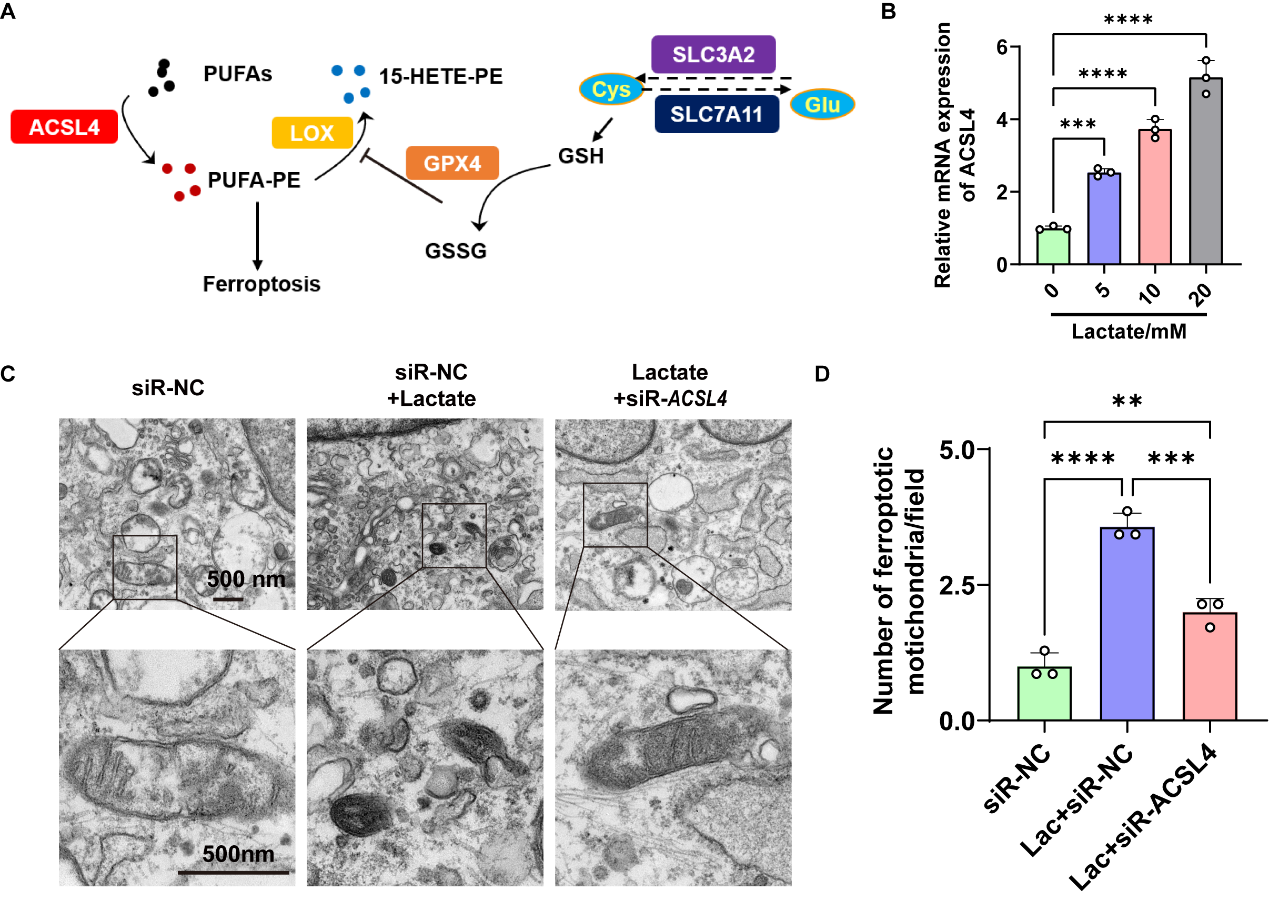


**Figure S6** A: Ilustration of the phospholipid peroxidation and related regulated genes during the initiation of ferroptosis. B: RT-qPCR result of the gene expression of ACSL4 in human NPCs treated by lactate in a dose-dependent manner (n=3). C and D: Representative images of TEM for mitochondrial structure in lactate-enriched NPCs with or without silencing ACSL4, as well as the quantitative result. Scale bar=500nm.


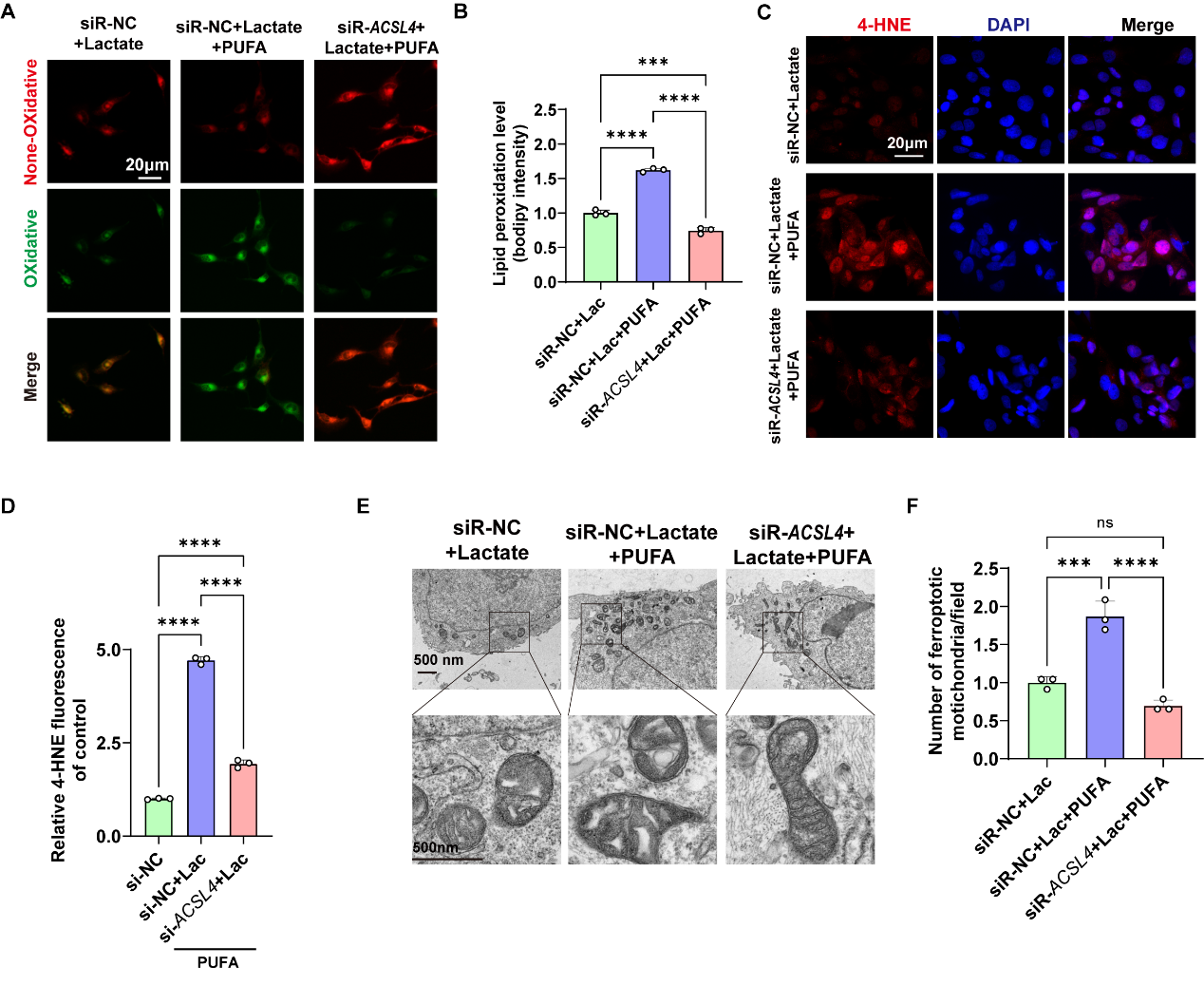


**Figure S7** A and B: After silencing *ACSL4*, intracellular LPO level was detected by confocal microscopy after BODIPY staining in lactate/PUFA-enriched NPCs, as well as the quantitative result (n=3). Scale bar=20μm. C and D: Representative image of IF staining for 4-HNE in in lactate/PUFA-enriched NPCs, as well as the quantitative result (n=3). E and F: Representative images of TEM for mitochondrial structure in lactate/PUFA-enriched NPCs, as well as the quantitative result (n=3). Scale bar=500nm. All data are shown as the mean ± SD. *P < 0.05, **P < 0.01, ***P < 0.001, ****P < 0.0001. ACSL4: Acyl-CoA Synthetase Long Chain Family Member 4; LPO: Lipid peroxidation; PUFA: Polyunsaturated fatty acids; NPCs: Nucleus pulposus.


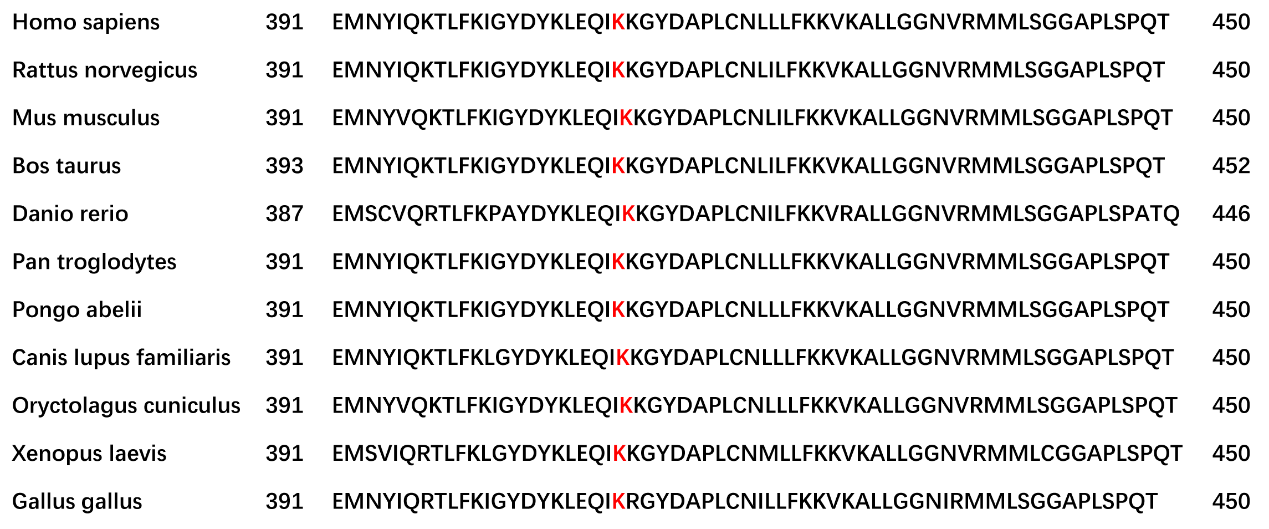


**Figure S8** The K412 site in ACSl4 is conserved. The sequences around ACSL4 K412 from different species were aligned. Conserved lysine residues corresponding to human ACSL4 K412 are marked in red.


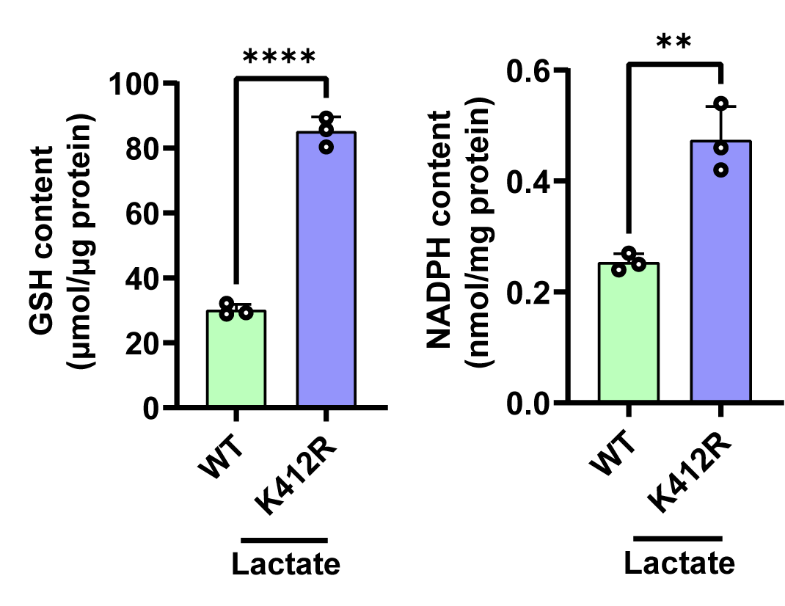


**Figure S9** Quantitative results of the level of GSH and NADPH in K412R site mutation and wild-type flag-ACSL4 overexpressed NPCs in the presence of lactate (n=3). All data are shown as the mean ± SD. *P < 0.05, **P < 0.01, ***P < 0.001, ****P < 0.0001. GSH: Glutathione; NADPH: Nicotinamide adenine dinucleotide phosphate.
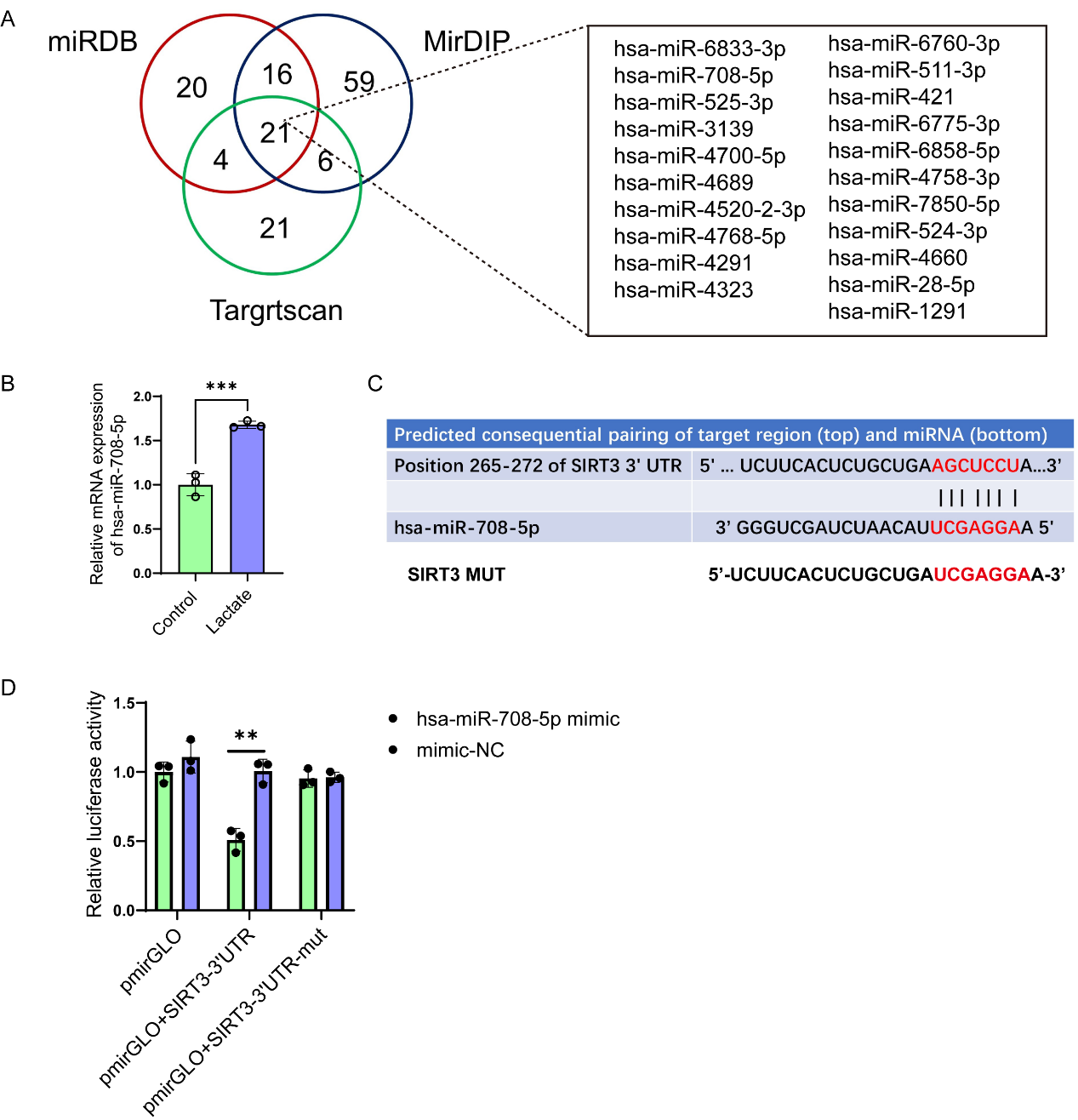


**Figure S10 miR-708-5p can be a potential bridge for lactate in regulating SIRT3.** A) Three different algorithms, including mitarget scan, mirDIP, and

miRDB were used to predict SIRT3 mRNA-binding miRNA candidates. B) miR-708-5p was assayed by qPCR after lactate treatment for 24 h in human NPCs. C) Sequence alignment between miR-708-5p, WT and MUT of 3’-UTR of SIRT3. D) Human NPCs were transiently co-transfected with either a WT or mutant SIRT3 3’UTR reporter plasmid and either a miR-708-5p mimics. The cells were harvested at 48h after transfection, and then luciferase activity was measured by dual-luciferase reporter assay. All data are shown as the mean ± SD. Statistical significance was determined by one-way ANOVA. **P < 0.01, ***P < 0.001.


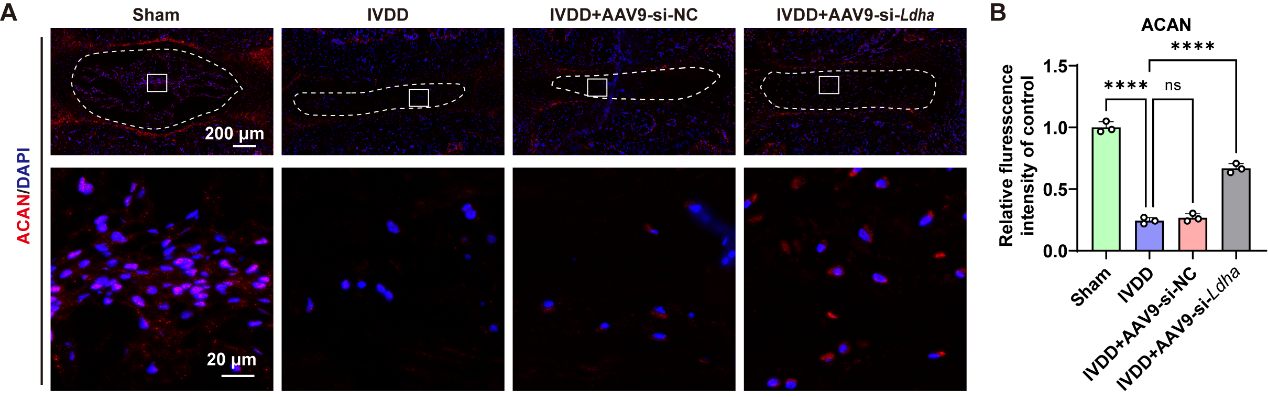


**Figure S11** A and B) Representative images and quantitative results of IF staining for ACAN of mice tail IVD tissue in sham, IVDD, IVDD+AAV9-siNC, and IVDD+AAV9-siLdha group, respectively.
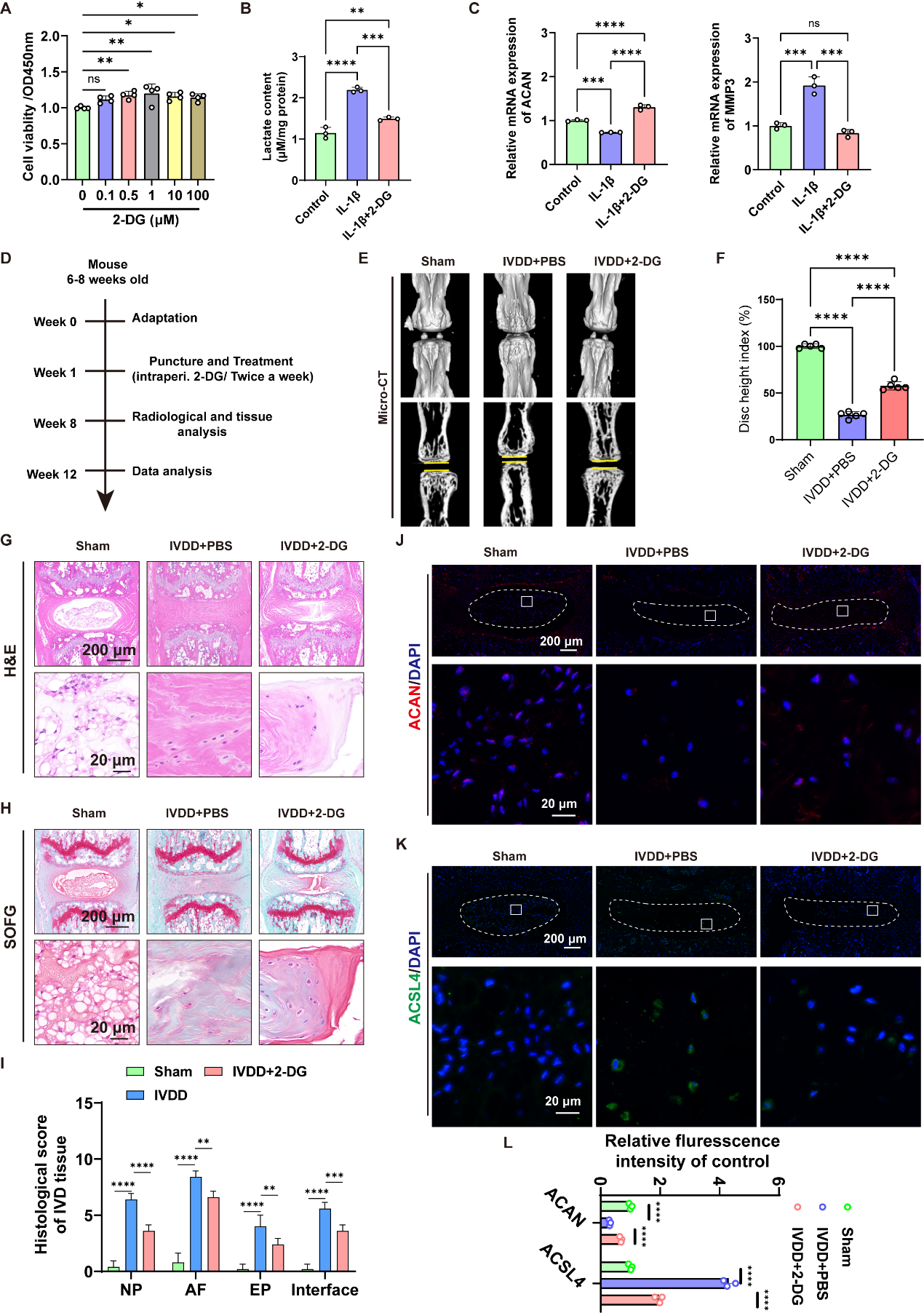


**Figure S12** **Inhibiting glycolysis via drug treatment ameliorates protein lactylation and ACSL4-induced ferroptosis and IVDD in mice** A) The effects of 2-DG on viability of human NPCs in a dose-dependent manner (n=4). B) The content of lactate in human NPCs treated by IL-1β with or without 2-DG (n=3). C) RT-qPCR results of the expression of *ACAN* and *MMP3* in human NPCs treated by IL-1β with or without 2-DG (n=3). D) Illustration of the animal experiment design to investigate the effect of decreasing lactate content via 2-DG on IVDD in vivo. E and F) Representative images of micro-CT in evaluating the disc height of mouse tail IVD in sham, IVDD+PBS, IVDD+2-DG group, respectively (n=5). G) Representative images of H&E staining of mice tail IVD tissue in sham, IVDD, and IVDD+2-DG group, respectively. H) Representative images of SOFG staining of mice tail IVD tissue in sham, IVDD, and IVDD+2-DG group, respectively. I) Histological score of mice tail IVD tissue in sham, IVDD, and IVDD+2-DG group, respectively (n=5). J) Representative images of IF staining for ACAN of mice tail IVD tissue in sham, IVDD, and IVDD+2-DG group, respectively. K) Representative images of IF staining for ACSL4 of mice tail IVD tissue in sham, IVDD, and IVDD+2-DG group, respectively. L) Quantitation of the IF staining for ACAN and ACSL4 (n=3). IVDD: Intervertebral disc degeneration; SOFG: Safranin O-Fast Green; H&E: Hematoxylin and eosin; ACAN: Aggrecan; MMP3: Matrix metalloprotease3; 2-DG: 2-Deoxy-D-glucose. All data are shown as the mean ± SD. *P < 0.05, **P < 0.01, ***P < 0.001, ****P < 0.0001.


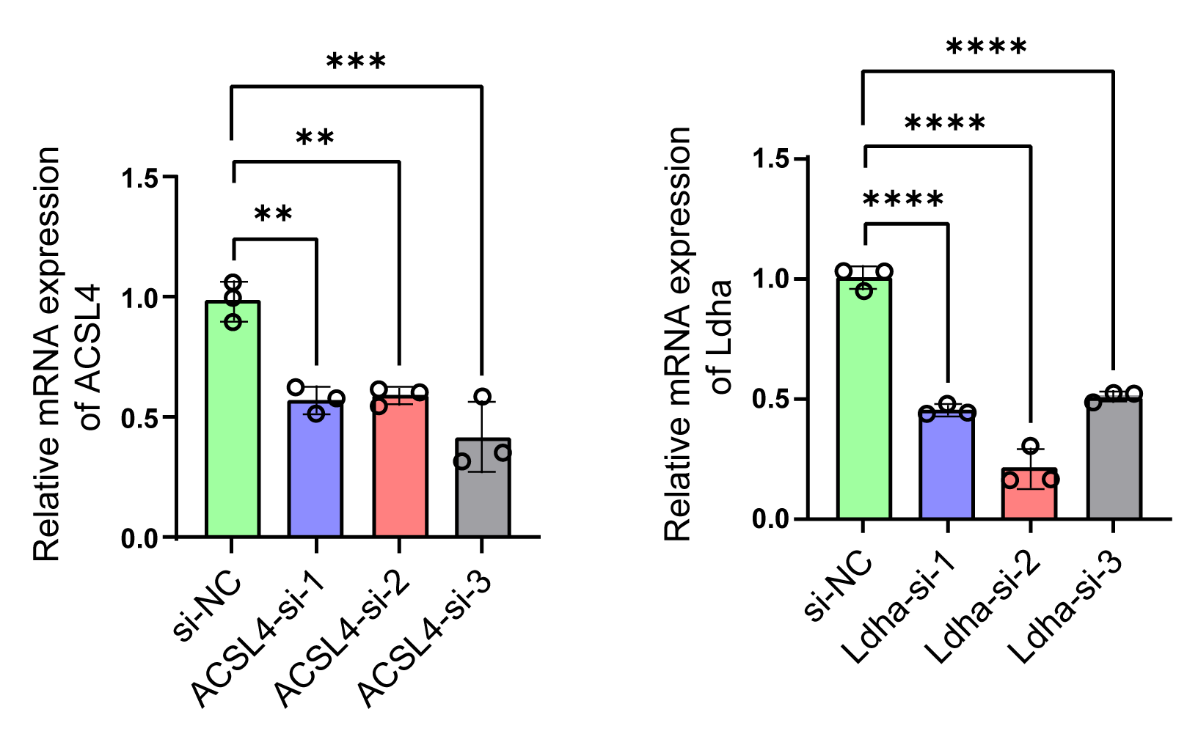
**Figure S13 The results of RT-qPCR for the selection of the best siRNA for human *ACSL4* and mouse *Ldha.***

**Table S1 Detailed information for patients that provided by the authors (Ji Tu et al. PMID: 34825784)**

| **Patient ID** | **S1** | **S2** | **S3** | **S4** | **S5** | **S6** | **S7** | **S8** |
| --- | --- | --- | --- | --- | --- | --- | --- | --- |
| **Age [years]** | 63 | 41 | 56 | 65 | 64 | 53 | 54 | 56 |
| **Gender** | Male | Male | Female | Female | Female | Female | Male | Male |
| **Weight [kg]** | 60 | 73.5 | 62 | 76 | 50 | 60 | 68 | 55 |
| **Reason for surgery** | Burst fracture | Burst fracture | Lumbar disc herniation | Lumbar disc herniation | Lumbar disc herniation | Lumbar disc herniation | Lumbar disc herniation | Lumbar disc herniation |
| **Pfirrmann grading** | II | II | III | III | IV | IV | V | V |
| **CRP [mg dL^−1^]** | 0.047 | 0.706 | 0.079 | 0.154 | 0.027 | 0.184 | 0.011 | 0.022 |
| **WBC (109 L^−1^)** | 6.46 | 11.37 | 7.25 | 6.78 | 4.83 | 5.53 | 10.79 | 5.75 |
| **Lymphocyte (109 L^−1^)** | 2.16 | 2.35 | 1.63 | 2.15 | 1.55 | 2.28 | 3.55 | 1.86 |
| **Monocyte (109 L^−1^)** | 0.64 | 1.02 | 0.47 | 0.54 | 0.19 | 0.49 | 0.83 | 0.61 |
| **Blood glucose [mmol L^−1^]** | 3.77 | 5.4 | 5.05 | 5.41 | 4.52 | 4.66 | 4.84 | 4.7 |
| **ALT [U L^−1^]** | 7.4 | 27.7 | 18.7 | 10.6 | 13.6 | 26.5 | 20.6 | 33.7 |
| **AST [U L^−1^]** | 12.7 | 13.4 | 14.8 | 12.3 | 12.6 | 18.3 | 18 | 17 |
| **Total protein [g L^−1^]** | 57.3 | 63.3 | 73.3 | 66.5 | 69 | 66.3 | 71.5 | 65.8 |
| **Serum albumin [g L^−1^]** | 37.4 | 41 | 44.9 | 42.1 | 44 | 40.7 | 46.3 | 39.4 |
| **BUN [mmol L^−1^]** | 3.3 | 5 | 4.8 | 6.1 | 5.5 | 4.6 | 4.9 | 6.4 |
| **Cre [μmoI L^−1^]** | 42.4 | 59.1 | 46.4 | 38 | 49.1 | 53.8 | 60.3 | 82 |
| **TT [s]** | 21.6 | 18.4 | 18.1 | 19.8 | 19.5 | 19.7 | 18.2 | 18.6 |
| **APTT [s]** | 26.7 | 22.8 | 21.9 | 24.4 | 26.2 | 22.4 | 23.6 | 25.9 |
| **INR** | 1.01 | 0.92 | 1.02 | 1.07 | 1 | 0.99 | 0.96 | 0.92 |

**Table S2. Patient’s information**

| No. | Admission number | Sex | Age | Diagnosis | Surgical  Level | Pfirrmann  score |
| --- | --- | --- | --- | --- | --- | --- |
| 1 | 865095 | F | 34 | Lumbar compressive fracture | L4/L5 | II |
| 2 | 861442 | F | 68 | Lumbar compressive fracture | L3/L4 | II |
| 3 | 864551 | M | 77 | Tethered cord syndrome | L4/L5 | II |
| 4 | 864552 | F | 70 | Lumbar spondylolysis | L4/L5 | II |
| 5 | 864649 | F | 82 | Tethered cord syndrome | L4/L5 | II |
| 6 | 864554 | F | 53 | Lumbar spondylolisthesis | L3/L4 | III |
| 7 | 821240 | M | 71 | Lumbar spinal stenosis | L5/S1 | III |
| 8 | 846565 | M | 53 | Lumbar spinal stenosis | L4/L5 | III |
| 9 | 827060 | F | 42 | Lumbar spinal stenosis | L4/L5 | III |
| 10 | 864562 | M | 49 | Lumbar spinal stenosis | L4/L5 | III |
| 11 | 468542 | F | 40 | Lumbar disc herniation | L5/S1 | IV |
| 12 | 831386 | M | 39 | Lumbar spinal stenosis | L4/L5 | IV |
| 13 | 864573 | F | 65 | Lumbar spinal stenosis | L3/L4 | IV |
| 14 | 864586 | M | 33 | Lumbar disc herniation | L4/L5 | IV |
| 15 | 861443 | F | 59 | Lumbar disc herniation | L5/S1 | IV |
| 16 | 864742 | F | 31 | Lumbar spondylolisthesis | L5/S1 | V |
| 17 | 865200 | F | 50 | Lumbar disc herniation | L4/L5 | V |
| 18 | 862059 | M | 18 | Lumbar disc herniation | L5/S1 | V |
| 19 | 865210 | F | 73 | Lumbar spinal stenosis | L4/L5 | V |
| 20 | 865261 | F | 65 | Lumbar spinal stenosis | L5/S1 | V |

**Table S3. Primer sequences for human tissue and cells**

| Gene name | Gene ID | Reference sequence | Primer Sequence (5'-3') |
| --- | --- | --- | --- |
| Human genes | | | |
| *ACAN* | 176 | NC_000015 | F: GGGAAGGCTGCTATGGAGAC  R: ACCTCACCCTCCATCTCCTC |
| *MMP3* | 4314 | NM_002422 | F: GGTGTGGAGTTCCTGATGTTGGTC  R: AGCCTGGAGAATGTGAGTGGAGTC |
| *MMP13* | 4322 | NM_002427 | F: ATCTGAACTGGGTCTTCCAA  R: GCCTGTATCCCTCAAAGTGAAC |
| *IL-1β* | 3553 | NM_000576 | F: GATATGGAGCAACAAGTGGT  R: AGGACAGGTACAGATTCTTTTC |
| *TNFα* | 7124 | NM_000594 | F: CCA GGC AGT CAG ATC ATC TTC  R: GCT TGA GGG TTT GCT ACA ACA |
| *ACSL4* | 2182 | NM_001318509 | F: AAG TAG ACC AAC GCC TTC A  R: CAG TCC AGG TAT TCT TTC ACA |
| *SIRT3* | 23410 | NM_001017524 | F: GCC TGT TTG CTG TGT TGA ACT  R: GGG ATT CCA GTT GGT CTG ATT |
| *β-ACTIN* | 60 | NM_001101 | F: AAGGTGACAGCAGTCGGTT  R: TGTGTGGACTTGGGAGAGG |
| Mouse genes | | | |
| *Ldha* | 50790 | NM_001136069 | F: CTT GTG TAG TGG TGA CCT GGT |
|  |  |  | R: AGT TGG CAG TGT GTC TCA GAG |
| *β-Actin* | 11461 | NM_007393 | F: CCT CTA TGC CAA CAC AGT |
|  |  |  | R: AGC CAC CAA TCC ACA CAG |

**Table S4 Histological grading system of human nucleus pulposus**

|  | **NP** **Features** | | | |  |
| --- | --- | --- | --- | --- | --- |
|  | **Cellularity** | | **Lesions** | **ECM** **structure** |  |
| **Sub** **Features included** | Single cells in lacunae, cell custers, necrosis,apoptosis, cell shrinkage, senescence (large cells), acellularity | | Tears, clefts and voids | Loss of Proteoglycan staining, mucoid degeneration, fibrosis, clear demarcation of the NP or presence of AF or CEP tissues in the NP |  |
| **Score** | **0** | Single cells in lacunae,no  evidence of necrotic,  apoptotic or senescent cells | Normal intact NP with intact ECM | Clear ECM structure, Eosin staining present throughout the NP, if using PG stain clear staining can be seen throughout the NP. Matrix structure does not contain lamella or ordered  collagens. Clear  demarcation seen at  junctions of NP and AF and NP and CEP |  |
|  | **1** | <25% Cells in custers in lacunae or multiple nuclei in lacunae, may be some evidence of apoptosis (nuclei condensation), cell shrinkage. | Micro fissures present within localised areas of the NP | May be some loss of demarcation and limited loss of Eosin staining in proximity to the NP cells. |  |
|  | **2** | 25-75%of cells in clusters, May be evidence of apoptosis (cell shrinkage), necrosis (cell debris) and cellular senescence (large irregular cells), may be areas of evidence cell loss (empty lacunae) | Micro fissures across the majority of the NP or may be localised larger clefts and voids | Presence of demarcation within the NP-noted by areas of structured collagens or fibrosis within the NP.Loss of Eosin staining around the proximity of cells but most matrix staining intact. |  |
|  | **3** | >75%in dusters,  evidence of empty  lacunae,or may be  evidence of apoptotic,  necrotic and senescent  cells,hypercellularity. | Presence of large clefts and voids within the NP and extensive fissures. | Substantial demarcation, or fibrosis,may be evidence of mucoid degeneration and loss of eosin staining which is more widespread than the immediate pericellular locations. |  |
|  |  |  |  |  |  |

NP: Nucleus pulposus; AF: Annulus fibrosus; ECM: Extracellular matrix

**Table S5 Histological grading system of mouse intervertebral disc**

| **Nucleus** **pulposus** **(Scoring** **range** **0-3)** | | | | |
| --- | --- | --- | --- | --- |
| **Features** | **0** | **1** | **2** | **3** |
| **NP** **cellularity** **& morphology** | -Stellate or spindle shaped NP cells with or without evidence of physaliferous/vacuolated cells -Evenly spread NP cells. | -Small and rounder NP cells. -Cluster of small NP cells. -Less than 50%NP cell loss. -Lacunae with a single NP cell. | -Multiple NP nuclei within a lacunae. -About 50 to 80%NP cell loss. | -Greater than 80%loss of cells in NP compartment. -Empty lacunae with no cells. |
| **NP** **fibrosis** | Absence of fibrosis. | Evidence of rare fibrous strands between NP cells. | Moderate fibrosis in NP compartment. | NP compartment filled with fibrous lamella/tissue. |
| **NP** **matrix organization** | Diffuse matrix evident between NP cells. | Mild consolidation of ECM between NP cells. | -Mild matrix disorganization. -Matrix granulation or dense clumps. | -Severe matrix disorganization. -Dense matrix clumps. |
| **Annulus fibrosus (Scoring range 0-3)** | | | | |
| **Features** | **0** | **1** | 2 | **3** |
| **AF** **cellularity** | Fibroblast or elliptical- shaped AF cells in all lamellae. | Round-shaped cells in less than one third of the inner AF lamellae. | Round-shaped cells in more than half of inner AF lamellae. | Absence of fibroblasts/elliptical cells and round cells present in all AF lamellae. |
| **AF** **bulging** | Absence of inward and outward protrusion of AF lamellae. | -Protrusion or bulging of AF lamellae into NP space. -Absence of outward bulging of AF lamellae. | -Inward protrusion of AF lamellae. -Outward bulging of AF lamellae. | Severe inward and outward bulging or protrusion of AF lamellae. |
| **AF** **lamellar organization** | Organized concentric AF lamellae. | Wavy AF fibers with mild lamellar disorganization. | -Widening of AF lamellae.  -Disorganization of AF lamellae. | Marked loss of normal AF lamella organization and structure. |
| **AF** **clefts/fissures** | Absence of clefts/fissuresin AF. | Mild cleft/fissures in outer AF. | Appearance of clefts or fissures between and across AF lamellae. | Several clefts or fissures through the depth of AF. |
| **End** **plate** **(Scoring** **range** **0-2)** | | | | |
| **Features** | **0** |  | **1** | **2** |
| **EP** **cellularity** | EP cells present in defined layers. |  | EP cells distributed in matrix and may not be organized in layers. | Empty lacunae with no EP cells. |
| **EP** **fissures/ micro-fractures** | Absence of micro-fractures/ fissures. |  | Presence of rare micro-fractures/ fissures between EP layers. | Presence of severe micro- fractures/fissures extending through the width of EP. |
| **Schmorl's** **node** | Absence of Schmorl's node. |  |  | Presence of Schmorl's node/s. |
| **Interface/boundary** **(Scoring** **range** **0-2)** | | | | |
| **Features** | **0** |  | 1 | **2** |
| **Cellularity** | Cells present within their respective compartments. |  | Presence of cells in lacunae at the boundary. | Presence of cells in lacunae with the source of compartment unclear. |
| **NP-AF boundary** | Defined boundary. |  | Mild inward fibrosis and merger of AF into NP. | Loss of NP-AF boundary. |
| **NP-EP** **boundary** | Defined boundary. |  | Mild fibrosis and merger of NP and EP. | Loss of NP-EP boundary. |
| **Disruption** **of** **AF lamella** **integration to** **EP** | Organized AF lamellae and integration into EP |  | Mild disruption of AF lamellae integration into EP. | Disruption of AF lamellae integration into EP with or without presence of tears and fissures. |

NP: Nucleus pulposus; AF: Annulus fibrosus; EP: Endplate; ECM: Extracellular matrix

**Table S6 The synthetic siRNA sequences for human *ACSL4*, human *SIRT3*, and mouse *Ldha***

| Species | Gene name | Sequences |
| --- | --- | --- |
| Human | *ACSL4-*si-1 | 5’- GCAGAGAUAUCUUGCUUUATT dTdT-3’  5’- UAAAGCAAGAUAUCUCUGCTTdTdT-3’ |
|  | *ACSL4-*si-2 | 5’- GCUGUGAAAUUAAGCUAAATTdTdT-3’  5’- UUUAGCUUAAUUUCACAGCTTdTdT-3’ |
|  | *ACSL4-*si-3 | 5’- GCAAAGAAGCAGUAGUUCATTdTdT-3’  5’- UGAACUACUGCUUCUUUGCTT dTdT-3’ |
| Human | *SIRT3*-si-1 | 5’- GGUGGAAGAAGGUCCAUAUTTdTdT-3’  5’- AUAUGGACCUUCUUCCACCTTdTdT-3’ |
|  | *SIRT3*-si-2 | 5’- GAAACUACAAGCCCAACGUTTdTdT-3’  5’- ACGUUGGGCUUGUAGUUUCTTdTdT-3’ |
|  | *SIRT3*-si-3 | 5’- CUUGCUGCAUGUGGUUGAUTTdTdT-3’  5’- AUCAACCACAUGCAGCAAGTTdTdT-3’ |
| Mouse | *Ldha*-si-1 | 5’-GAACAAGAUUACAGUUGUUdTdT-3’  5’-AACAACUGUAAUCUUGUUCdTdT-3’ |
|  | *Ldha*-si-2 | 5’-AGCAAAGACUACUGUGUAAdTdT-3’  5’-UUACACAGUAGUCUUUGCUdTdT-3’ |
|  | *Ldha*-si-3 | 5’-CAGUGGAUAUCUUGACCUAdTdT-3’  5’-UAGGUCAAGAUAUCCACUGdTdT-3’ |

**Table S7 The list of the abbreviations in this study**

| **Abbreviation** | **Full name** |
| --- | --- |
| IVDD | Intervertebral disc degeneration |
| NPCs | Nucleus pulposus cells |
| AAV9 | Adenoviral‐associated viruses 9 |
| ACSL4 | Acyl-CoA Synthetase Long Chain Family Member 4 |
| SIRT3 | Sirtuin-3 |
| 2-DG | 2-Deoxy-D-glucose |
| LBP | Low back pain |
| ECM | Extracellular matrix |
| MMPs | Matrix metalloproteases |
| ADAMTSs | A Disintegrin and Metalloproteinase with Thrombospondin motifs |
| OA | Osteoarthritis |
| GLUT1 | Glucose transporter 1 |
| AGEs | Advanced glycation end products |
| HIF-1α | Hypoxia-inducible factor-1α |
| PCA | Principal component analysis |
| UMAP | Uniform manifold approximation and projection |
| DEGs | Differentially expressed genes |
| GO | Gene ontology |
| KEGG | Kyoto Encyclopedia of Genes and Genomes |
| mTOR | Mammalian target of rapamycin |
| ATP | Adenosine triphosphate |
| GAPDH | Glyceraldehyde-3-phosphate dehydrogenase |
| LDHA | Lactate dehydrogenase A |
| HK2 | Hexokinase 2 |
| ENO1 | Enolase 1 |
| PKM | Pyruvate kinase M |
| G6PD | Glucose-6-phosphate dehydrogenase |
| PFK | Phosphofructokinase |
| PGK1 | Phosphoglycerate Kinase 1 |
| MCT4 | Monocarboxylate transporter 4 |
| IL-1β | Interleukin 1β |
| TNFα | Tumor necrosis factor-α |
| ECAR | Extracellular acidification rate |
| OCR | Oxygen consumption rate |
| ACAN | Aggrecan |
| COL2A1 | Type 2 collagen |
| H&E | Hematoxylin and eosin |
| SOFG | Safranin O-Fast Green |
| LPO | Lipid peroxidation |
| ROS | Reactive oxygen species |
| GSH | Glutathione |
| NADPH | Nicotinamide adenine dinucleotide phosphate |
| SOD | Superoxide Dismutase |
| MDA | Malondialdehyde |
| Fer-1 | Ferrostatin-1 |
| PUFA | Polyunsaturated fatty acids |
| MKI67 | Marker of proliferative protein Ki-67 |
| SERPINE1 | Serpin family E member 1 |
| ATF4 | Activating transcription factor 4 |
| SLC38A1 | Solute carrier family 38 member 1 |
| COL1A1 | Type 1 collagen |
| PARP10/15 | Poly(ADP-ribose) polymerase family member 10/15 |
| TMBIM4 | Transmembrane Bax inhibitor-1 motif-containing 4 |
| ANO6 | Anoctamin 6 |
| LIFR | Leukemia inhibitory factor receptor |
| MAP1LC3A | Microtubule-associated protein 1 light chain 3 alpha |
| GJA1 | Gap junction protein alpha1 |
| CDKN2A | cyclin-dependent kinase inhibitor 2 |
| MYB | Myeloblastosis |
| 4-HNE | 4-hydroxy-2-nonenal |
| PTMs | Post-translational modifications |
| WT | Wild type |
| HDAC1-3 | Histone deacetylase 1-3 |
| AF | Annulus fibrosus |
| GAG | Glycosaminoglycan |
| ScRNA-seq | Single-cell RNA sequencing |
| OXPHOS | Oxidative phosphorylation |
| TAC | Tricarboxylic acid cycle |
| TIGAR | TP53-induced glycolysis and apoptosis regulator |
| Kla | Lysine lactylation |
| GEO | Gene Expression Omnibus |
| MRI | Magnetic resonance imaging |
| RT-qPCR | RNA reverse transcription, and quantitative real-time polymerase chain reaction |
| GSEA | Gene set enrichment analysis |
| TEM | Transmission electron microscopy |
| HPLC | High-performance liquid chromatography |
| IHC | Immunohistochemistry |
| IF | Immunofluorescence |
| SD | Standard deviation |
| TEM | Transmission electron microscopy |
